# Reduced-energy diet in women with gestational diabetes: the dietary intervention in gestational diabetes DiGest randomized clinical trial

**DOI:** 10.1038/s41591-024-03356-1

**Published:** 2025-02-19

**Authors:** Laura C. Kusinski, Danielle Jones, Nooria Atta, Elizabeth Turner, Suzanne Smith, Linda M. Oude Griep, Kirsten Rennie, Emanuella De Lucia Rolfe, Stephen J. Sharp, Vern Farewell, Helen R. Murphy, Roy Taylor, Claire L. Meek

**Affiliations:** 1 Leicester Diabetes Centre and Leicester NIHR Biomedical Research Centre, University of Leicester, Leicester General Hospital, Leicester, UK; 2 Institute of Metabolic Science – Medical Research Laboratories, Cambridge Biomedical Campus, University of Cambridge, Cambridge, UK; 3 MRC Epidemiology Unit, University of Cambridge School of Clinical Medicine, Institute of Metabolic Science, Cambridge Biomedical Campus, Cambridge, UK; 4 MRC Biostatistics Unit, Cambridge Biomedical Campus, Cambridge, UK; 5 University of East Anglia, Bob Champion Building, Norwich Research Park, Norwich, UK; 6 Norfolk and Norwich University Hospitals NHS Foundation Trust, Norwich, UK; 7 Newcastle Magnetic Resonance Centre, Translational and Clinical Research Institute, Newcastle University, Health Innovation Neighbourhood, Newcastle Upon Tyne, UK; 8 Cambridge Universities NHS Foundation Trust, Cambridge Biomedical Campus, Cambridge, UK; 9 University Hospitals of Leicester NHS Trust, Leicester General Hospital, Leicester, UK

**Keywords:** Endocrine system and metabolic diseases, Endocrine system and metabolic diseases, Reproductive biology

## Abstract

Reduced-energy diets promote weight loss and improve long-term outcomes in type 2 diabetes but are untested in gestational diabetes. We aimed to identify if weight loss in pregnancy improves perinatal outcomes in gestational diabetes. We performed a multicentre parallel, randomized, controlled, double-blind trial of energy restriction in women with singleton pregnancies, gestational diabetes and body mass index ≥25 kg m^−^^2^. Participants were randomized to receive a standard-energy control diet (2,000 kcal d^−1^) or reduced-energy intervention diet (1,200 kcal d^−1^) from enrollment (29 weeks) until delivery, provided as weekly diet boxes (40% carbohydrate, 35% fat, 25% protein). The randomization was performed in a 1:1 ratio, stratified by center and blinded to the participants and study team. Primary outcomes were maternal weight change from enrollment to 36 weeks and offspring birth weight. In total, 425 participants were randomized to the control (*n* = 211) or intervention (*n* = 214). Outcome data were available for 388 of 425 (90.1%) participants at 36 weeks and 382 of 425 (89.8%) at delivery. There was no evidence of a difference in maternal weight change to 36 weeks between groups (intervention effect −0.20 (95% confidence interval −1.01, 0.61); *P* > 0.1) and offspring standardized birth weight (intervention effect 0.005 (−0.19, 0.20); *P* > 0.1). A reduced-energy diet was safe in pregnancy. ISRCTN registration no. 65152174.

## Abstract (web summary)

In a clinical trial in pregnant women with gestational diabetes, a dietary intervention for energy restriction was safe and did not result in any difference in maternal weight or offspring standardized birth weight outcomes compared to the control arm.

## Main

Gestational diabetes affects 6–15% of pregnancies internationally and is associated with suboptimal maternal and offspring outcomes^1^. Risk factors for gestational diabetes include overweight and obesity^2^ and excess weight gain during pregnancy^3^. Medical nutritional therapy is a foundational aspect of gestational diabetes management, but little evidence exists regarding optimal energy intake for affected patients^4^. The role of dietary weight loss in the management of women with gestational diabetes is unknown.

In non-pregnant people with type 2 diabetes, weight loss improves glycaemia and reduces medication requirements, leading to remission of clinical diabetes^5,6^. Several dietary strategies have been successfully used to support weight loss in this population, including very-low-energy diets (400–500 kcal d^−1^), low-energy diets (1,000–1,500 kcal d^−1^) and formula meal replacements^7^. A similar approach may be valuable in patients with gestational diabetes, who are at increased risk of type 2 diabetes, but energy restriction has not been advocated or widely tested in pregnancy.

Current international guidelines for weight change in pregnancy were developed for healthy pregnant women and have not been customized for women with diabetes or obesity who are at increased risk of perinatal complications. The Institute of Medicine guidelines (now called the National Academy of Medicine) are based upon a woman’s prepregnancy body mass index (BMI)^8^. Women are recommended to gain 11.4–15.0 kg, 6.8–11.3 kg and 5.0–9.1 kg for those with a prepregnancy BMI in the normal weight, overweight and obese ranges respectively^9^. However, several recent retrospective cohort studies have identified that weight gain below the guidelines, or even weight loss, may improve pregnancy outcomes in women with prepregnancy obesity or gestational diabetes^9–11^. Despite the mounting evidence favoring reduced gestational weight gain in women with gestational diabetes, there are very few intervention studies that have successfully addressed gestational weight gain in this population.

We performed a randomized controlled double-blind trial using a whole-diet intervention to assess pregnancy outcomes after energy restriction in women with gestational diabetes.

## Results

### Patient disposition

**Fig. 1:**
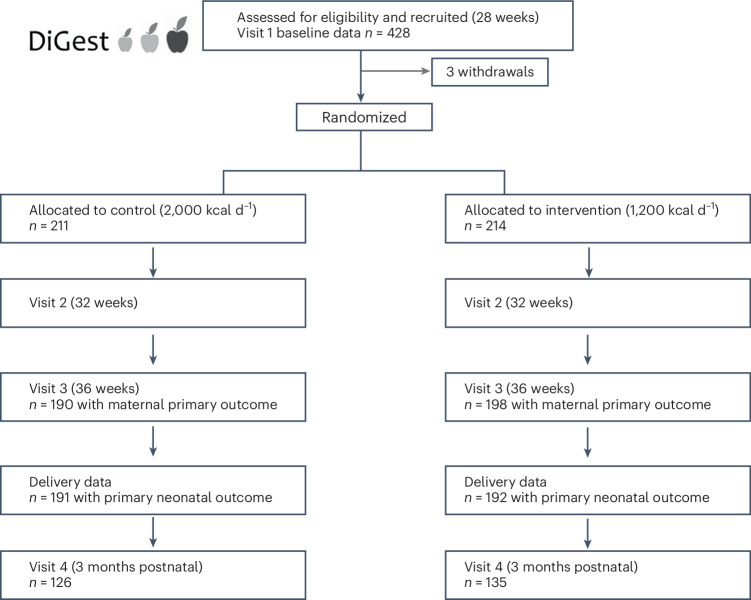
Flow chart of DiGest study participants. Participants were randomized to a trial arm after baseline measurements were complete and details of numbers achieved with maternal and neonatal primary outcomes.

**Extended Data Fig. 1:**
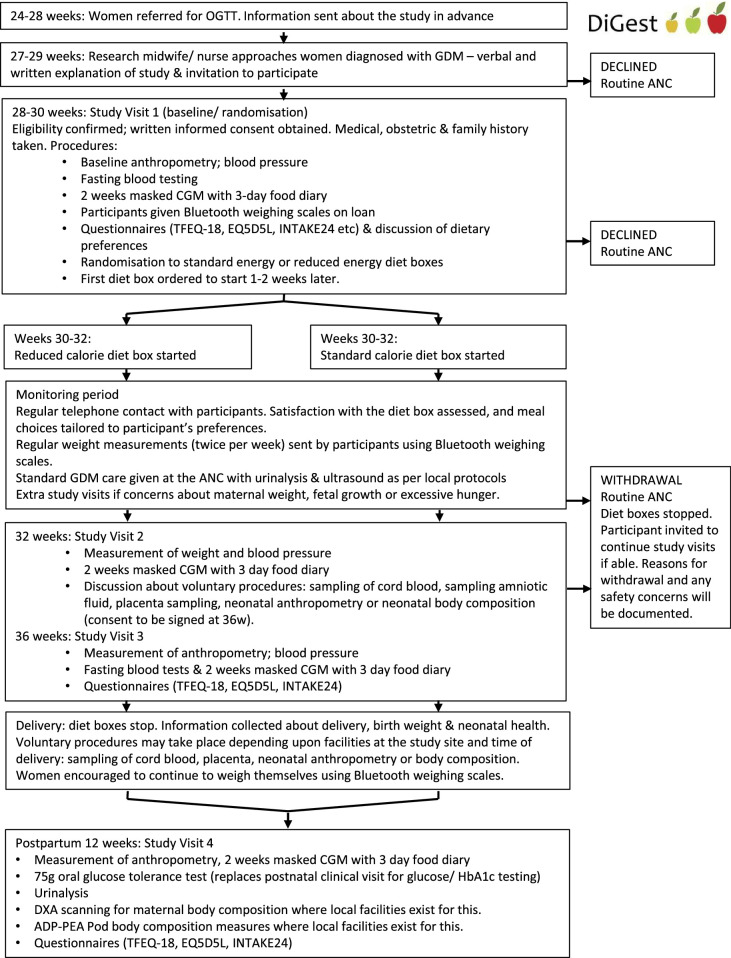
The design of the DiGest randomized controlled trial^32^. Overview of the study protocol detailing study recruitment process, study timeline and the measurements taken at each study visit.

From November 2019 to July 2023, 428 participants were enrolled at eight centers in England and randomized to receive a control diet (*n* = 211; standard energy content, 2,000 kcal d^−1^) or intervention diet (*n* = 214; energy restriction, 1,200 kcal d^−1^ (Fig. 1 and Extended Data Fig. 1). Overall, the two groups were balanced with respect to baseline characteristics (Table 1). Characteristics of participants with missing data for maternal (*n* = 38) or neonatal (*n* = 45) primary endpoints were similar to those of the trial population overall (Extended Data Tables 1 and 2).

**Table 1:** Baseline characteristics of patients at enrollment

	*n*	All participants *n* = 425	*n*	Control *n* = 211	*n*	Intervention *n* = 214
Maternal age (years)	425	33.03 (5.04)	211	32.80 (5.11)	214	33.26 (4.97)
BMI (kg m^−^^2^)	425	35.67 (6.44)	211	36.04 (6.72)	214	35.30 (6.15)
Self-reported ethnicity	425		211		214	
White		332 (78.12)		163 (77.25)		169 (78.97)
Asian		73 (17.18)		40 (18.96)		33 (15.42)
Black		17 (4.00)		6 (2.84)		11 (5.14)
Other ethnic groups		3 (0.71)		2 (0.95)		1 (0.47)
Primiparous	385	136 (35.32)	192	61 (31.77)	193	75 (38.86)
Gestational weight gain pre-enrollment (kg)	424	3.94 (5.89)	211	3.80 (6.33)	213	4.09 (5.44)
Maternal education (>degree)	425	201 (47.29)	211	95 (45.02)	214	106 (49.47)
Index of multiple deprivation decile	412	6.53 (2.47)	204	6.50 (2.56)	208	6.56 (2.38)
Gestational diabetes in previous pregnancy	424	122 (28.77)	211	68 (32.23)	213	54 (25.35)
Health at enrollment						
Smoking	422	44 (10.43)	210	32 (15.24)	212	12 (5.66)
Physical activity PAEE (kJ kg^−^^1 ^d^−^^1^)	230	19.86 (12.65)	117	18.82 (11.68)	113	20.96 (13.55)
Habitual energy intake (kcal d^−1^)	223	1,570.92 (665.99)	114	1,555.77 (652.66)	109	1,586.77 (682.31)
Basal metabolic rate (J h^−1 ^kg^−^^1^)	385	1,643.07 (227.58)	192	1,645.66 (218.69)	193	1,640.49 (236.64)
Systolic blood pressure (mmHg)	418	115.69 (12.47)	208	114.97 (12.42)	210	116.41 (12.51)
Diastolic blood pressure (mmHg)	418	69.29 (10.12)	208	68.64 (10.33)	210	69.94 (9.86)
Diagnosis						
Gestational age at diagnosis	414	22.85 (6.40)	204	22.73 (6.54)	210	22.97 (0.97)
OGTT 0 h glucose (mmol l^−^^1^)	206	5.01 (0.71)	98	5.05 (0.76)	108	4.97 (0.65)
OGTT 2 h glucose (mmol l^−^^1^)	207	8.11 (1.67)	100	8.07 (1.81)	107	8.14 (1.52)
HbA1c (mmol mol^−^^1^)	147	39.00 (4.63)	76	39.99 (4.91)	71	39.01 (4.33)
HbA1c (%)	147	5.72 (0.42)	76	5.72 (0.50)	71	5.72 (0.40)
Medication use at enrollment						
Metformin	425	94 (22.12)	211	53 (25.12)	214	41 (19.16)
Short-acting insulin	425	38 (8.94)	211	15 (7.11)	214	23 (10.75)
Long-acting insulin	425	101 (23.76)	211	46 (21.80)	214	55 (25.70)
		All participants		Control		Intervention
	*n*	*n* = 425	*n*	*n* = 211	*n*	*n* = 214
Glycaemia at enrollment						
Days of CGM use	361	5.79 (2.24)	172	5.76 (2.24)	189	5.82 (2.23)
Mean CGM glucose (mmol l^−^^1^)	361	5.77 (0.77)	172	5.82 (0.67)	189	5.72 (0.85)
Mean CGM glucose (mg dl^−^^1^)	361	103.95 (13.89)	172	104.92 (11.98)	189	103.07 (15.40)
TIR (3.5–6.7 mmol l^−^^1^) (%)	361	77.02 (18.40)	172	76.44 (17.69)	189	77.55 (19.05)
		**83.30 (70.95–89.16)**		**82.74 (69.14–87.65)**		**84.09 (72.09–90.70)**
TAR (3.5–6.7 mmol l^−^^1^) (%)	361	21.32 (19.18)	172	22.24 (18.32)	189	20.48 (19.94)
		**15.05 (7.87–28.61)**		**15.68 (9.72–30.04)**		**14.25 (6.11–26.63)**
TBR (3.5–6.7 mmol l^−^^1^) (%)	361	1.66 (2.91)	172	1.32 (1.98)	189	1.97 (3.52)
		**0.53 (0.00–1.81)**		**0.43 (0.00–1.74)**		**0.66 (0.04–2.09)**
TIR (3.5–7.8 mmol l^−^^1^) (%)	361	90.80 (10.98)	172	91.48 (9.32)	189	90.18 (12.29)
		**94.46 (88.79–97.31)**		**94.70 (88.82–97.31)**		**94.10 (88.71–97.26)**
TAR (3.5–7.8 mmol l^−^^1^) (%)	361	7.54 (11.30)	172	7.20 (9.60)	189	7.85 (12.67)
		**3.20 (1.17**–**8.91)**		**3.21 (1.48**–**9.53)**		**3.20 (0.81**–**7.84)**
TBR (3.5–7.8 mmol l^−^^1^) (%)	361	1.66 (2.91)	172	1.32 (1.98)	189	1.97 (3.52)
		**0.53 (0.00**–**1.81)**		**0.43 (0.00**–**1.74)**		**0.66 (0.04**–**2.09)**
CV	361	18.22 (3.84)	172	17.90 (3.80)	189	18.51 (3.86)
s.d.	361	1.05 (0.29)	172	1.04 (0.26)	189	1.06 (0.31)

Results are presented as mean (s.d.) or *n* (%) or median (IQR) in bold as appropriate. Significance testing—linear or logistic regression adjusted by site. CV, coefficient of variation; PAEE, physical activity energy expenditure; TAR, time above range; TBR, time below range.

During the trial, 59 participants withdrew from the study (29 (13.7%) from control group; 30 (14.0%) from intervention group). A further 53 patients stopped receiving the diet boxes before delivery but remained in the study (13.3% control group; 11.7% intervention group). This occurred typically after 36 weeks (after collection of maternal endpoint data) and the reasons included participants growing tired of the food in light of impending delivery (<1–2 weeks); pregnancy complications such as preeclampsia or threatened preterm delivery, especially if requiring hospitalization; hunger; concern about high glucose concentrations and stress (Extended Data Table 3).

Participants received the dietary intervention for a mean of 6.15 (s.d. 3.24) weeks in the control arm and 6.35 (3.29) weeks in the intervention arm (Extended Data Table 4). Allowing for the 10-day period of baseline data collection, the mean number of eligible weeks between enrollment and delivery was 8.95 (s.d. 1.80) in the control and 9.27 (1.85) in the intervention group (Extended Data Table 4), giving ordering rates of 68.9% in the control group and 68.8% in the intervention group. Satisfaction levels were consistent throughout the trial, with most participants being highly satisfied or satisfied with the quality of the food (85% control group; 81% intervention group; Supplementary Table 1).

### Primary outcomes

There was no evidence of a difference in maternal weight change at 36 weeks, the primary maternal outcome, between groups (intervention +0.39 kg (4.23), control +0.54 kg (4.17); baseline-adjusted difference intervention versus control, *β* (adjusted effect size) −0.20 (95% confidence interval (CI) −1.02, 0.61); *P* = 0.623; Table 2). Results were unaffected when participants with preterm deliveries were included with adjustment for gestational age at delivery, when using multiple imputation (Extended Data Tables 5 and 6) and when the analysis was restricted to women who had ordered the diet boxes for 4 weeks or more (Extended Data Table 7).

**Table 2:** Primary outcomes summarized as mean and s.d. or median and IQR

	*n*	Control *n* = 211	*n*	Intervention *n* = 214	Intervention effect (95% CI)	*P*
Neonatal primary outcome						
Standardized birth weight (Intergrowth)	190	0.44 (0.91)	192	0.45 (1.04)	0.005 (−0.19, 0.20)	0.96
		**0.40 (−0.09–0.97)**		**0.46 (−0.22–1.12)**		
Detail						
Unadjusted					0.01 (−0.19, 0.21)	0.92
Incorporating stratification variable (study center)					0.005 (−0.19, 0.20)	0.96
Also adjusted for baseline					Not applicable	
Maternal primary outcome						
Weight change (kg)	190	0.54 (4.17)	198	0.39 (4.23)	−0.20 (−1.01, 0.61)	0.63
		**1.15 (−1.20–2.50)**		**0.35 (−1.70–2.30)**		
Detail						
Unadjusted					−0.15 (−0.98, 0.69)	0.73
Incorporating stratification variable (study center)					−0.17 (−0.99, 0.65)	0.68
Also adjusted for baseline weight					−0.20 (−0.01, 0.61)	0.63
Weight at enrollment (kg)	211	96.16 (20.25)	213	94.61 (19.94)		
Weight at 36 weeks (kg)	190	96.22 (19.41)	198	95.43 (19.88)		

Intervention effect is the baseline-adjusted difference in mean outcome between intervention and control groups, estimated from a linear regression model that also includes study center. Only prespecified primary analysis have regression results included; other results are given for context only. Median and IQR are shown in bold.

No significant difference was observed in the primary neonatal outcome, standardized birth weight (Intergrowth), between the intervention and control groups (0.45 (1.04) versus 0.44 (0.91); *β* 0.005 (95% CI −0.19, 0.20); *P* = 0.962; Table 2).

### Secondary outcomes

**Fig. 2:**
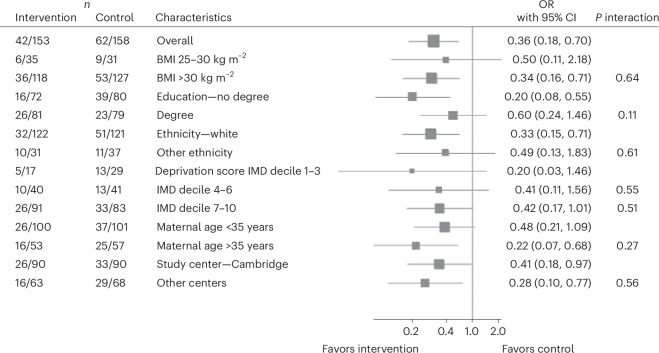
Interaction of maternal characteristics on the effect of the intervention on the requirement for long-acting insulin. Post hoc subgroup analysis to determine the interaction effect of participant characteristics on the effectiveness of the intervention for the requirement of long-acting insulin. Subgroup analyses were conducted for maternal baseline BMI, education, ethnicity, socioeconomic status, maternal age and study center. Estimated effect sizes were calculated using unadjusted logistic regression and are shown as ORs for each subgroup with 95% CIs. Interaction *P* values (*P* interactions > 0.05 for all subgroups) indicate no statistically significant interaction with any subgroup. IMD, Index of Multiple Deprivation. *n* represents the number of participants with that characteristic out of the total number of participants in that trial arm that required long-acting insulin.

Provision of a reduced-energy diet reduced requirements for long-acting insulin therapy (39.2% control, 27.5% intervention; odds ratio (OR) 0.36 (95% CI 0.18–0.70); *P* = 0.003; number needed to treat (NNT) 8.5) at 36 weeks (Table 3). The effect of the intervention on long-acting insulin requirements was not affected by maternal BMI at enrollment, education, ethnicity, deprivation score, maternal age or study center (Fig. 2). There was no evidence of differences in requirements for metformin or short-acting prandial insulin, delivery modality, blood pressure or continuous glucose monitoring metrics at 36 weeks between trial arms (Table 3). Postnatal hemoglobin A1c (HbA1c) was significantly lower in the intervention group after adjustment for baseline HbA1c and the study center: median HbA1c (control group (*n* = 36) interquartile range (IQR)) 40.0 (36.5–42.0) mmol mol^−^^1^; intervention group (*n* = 27) 37.0 (37.0–40.0) mmol mol^−^^1^. In percentage: control group 5.8 (5.5–6.0)%; intervention group 5.5 (5.5–5.8)%; *β* −2.36 mmol mol^−^^1^ (95% CI −4.46, −0.26); *P* = 0.029; −0.22% (95% CI −0.41, −0.02); *P* = 0.029 (Table 3). Outcomes from the core outcome set for diabetes in pregnancy are provided (Table 3 and Supplementary Table 2). Maternal-health-related quality of life was stable throughout the study (Supplementary Table 3).

**Table 3:** Secondary outcomes summarized as mean and s.d. or median and IQR

	*n*	Control *n* = 211	*n*	Intervention *n* = 214	Intervention effect (95% CI)	*P*
Neonatal secondary outcomes						
Birth weight (g)	191	3276.45 (442.23)	192	3289.77 (508.51)	11.71 (−73.95, 97.37)	0.79
Birth weight (Intergrowth centile)	190	62.99 (25.67)	192	62.50 (28.02)	−0.70 (−6.13, 4.73)	0.80
Birth weight (GROW centile)	191	44.60 (28.07)	192	45.00 (31.02)	0.25 (−5.72, 6.23)	0.94
Large for gestational age (Intergrowth)	190	35 (18.42)	192	39 (20.31)	OR; 1.11 (0.66, 1.86)	0.70
NICU admission	191	17 (8.90)	191	23 (12.04)	OR; 1.36 (0.69, 2.68)	0.38
Estimated gestation at birth (weeks)	192	38.45 (1.27)	192	38.42 (1.31)	−0.02 (−0.28, 0.23)	0.86
Cord blood C-peptide (umol l^−^^1^)	54	300.96 (211.18)	44	234.59 (189.82)	−61.68 (−142.38, 19.02)	0.13
Maternal secondary outcomes						
Cesarean section	211	98 (46.45)	214	84 (39.25)	OR; 0.74 (0.50, 1.09)	0.13
Metformin at 36 weeks	157	48 (30.57)	153	40 (26.14)	OR; 1.07 (0.58, 2.00)	0.82
Short-acting insulin at 36 weeks	157	24 (15.29)	153	17 (11.11)	OR; 0.43 (0.17, 1.08)	0.07
Long-acting insulin at 36 weeks	158	62 (39.24)	153	42 (27.45)	OR; 0.36 (0.18, 0.70)	0.003
TIR (3.5–6.7 mmol l^−^^1^) at 36 weeks (%)	112	75.32 (18.81)	115	74.93 (18.55)	−1.69 (−6.05, 2.66)	0.45
		**81.04 (64.52–88.89)**		**78.66 (65.67–87.62)**		
TIR (3.5–7.8 mmol l^−^^1^) at 36 weeks (%)	112	90.15 (11.36)	115	89.64 (10.03)	−0.23 (−2.78–2.31)	0.86
		**94.24 (86.97–97.14)**		**92.82 (85.83–96.53)**		
CGM mean glucose at 36 weeks (mmol l^−^^1^)	112	5.81 (0.81)	115	5.79 (0.79)	0.06 (−0.13, 0.25)	0.55
CGM mean glucose at 36 weeks (mg dl^−^^1^)	112	104.70 (14.63)	115	104.25 (14.18)	−1.02 (−2.39, 4.44)	0.55
Systolic blood pressure (mmHg)	155	118.36 (13.18)	165	117.90 (13.22)	−0.96 (−3.50, 1.58)	0.46
Diastolic blood pressure (mmHg)	155	70.92 (10.00)	165	72.40 (10.50)	0.77 (−1.27, 2.81)	0.46
Maternal outcomes at 3 months postnatally						
HbA1c (mmol mol^−^^1^)	36	36.55 (4.62)	27	36.99 (3.30)	−2.36 (−4.46, −0.26)	0.029
Change in HbA1c (mmol mol^−^^1^)	36	0.36 (5.8)	27	−0.67 (3.41)	−1.8 (−4.62, 1.02)	0.21
Baseline HbA1c (mmol mol^−^^1^)	36	38.7 (6.1)	27	38.9 (4.0)		
		**40.0 (34.5–42.0)**		**40.0 (35.0–42.0)**		
Postnatal HbA1c (mmol mol^−^^1^)	36	39.1 (5.8)	27	38.2 (2.6)		
		**40.0 (36.5–42.0)**		**37.0 (37.0–40.0)**		
HbA1c (%)	36	5.50 (0.42)	27	5.54 (0.30)	−0.22 (−0.41, −0.02)	0.029
Change in HbA1c (%)	36	0.03 (−2.01, 1.28)	27	−0.06 (−0.64, 0.55)	−0.16 (−0.42, 0.09)	0.21
Baseline HbA1c (%)	36	5.69 (0.6)	27	5.71 (0.4)		
		**5.8 (5.3–6.0)**		**5.8 (5.4–6.0)**		
Postnatal HbA1c (%)	36	5.72 (0.5)	27	5.65 (0.2)		
		**5.8 (5.5–6.0)**		**5.5 (5.5–5.8)**		
TIR (3.9–10.0 mmol l^−1^) (%)	98	97.22 (5.11)	99	97.39 (4.73)	0.20 (−1.31, 1.71)	0.80
		**99.02 (97.45–99.73)**		**98.70 (96.81–99.77)**		
CGM mean glucose (mmol l^−^^1^)	98	6.19 (0.71)	99	6.36 (0.77)	0.19 (−0.02, 0.41)	0.08
CGM mean glucose (mg dl^−^^1^)	98	111.56 (12.79)	99	114.58 (13.91)	3.49 (−0.37, 7.34)	0.08
Maternal weight (kg)	126	88.30 (18.67)	135	86.95 (18.31)	−0.45 (−1.89, 0.99)	0.54
Maternal BMI (kg m^−^^2^)	126	32.91 (6.09)	135	32.50 (6.27)	−0.13 (−0.66, 0.41)	0.64
Systolic blood pressure (mmHg)	119	118.56 (13.65)	128	119.14 (13.42)	0.50 (−2.60, 3.60)	0.75
Diastolic blood pressure (mmHg)	119	80.51 (11.96)	128	79.59 (13.87)	−1.58 (−4.68, 1.52)	0.32
Safety outcomes						
Small for gestational age (Intergrowth)	190	7 (3.68)	192	10 (5.21)		
Stillbirth	211	1 (0.47)	214	0 (0.00)		
Neonatal death	211	1 (0.47)	214	0 (0.00)		
Maternal death	211	1 (0.47)	214	0 (0.00)		
Congenital anomaly^a^	191	1 (0.47)	192	2 (1.04)		

For continuous outcomes, intervention effect is the baseline (where available)-adjusted difference in mean outcome between intervention and control groups, estimated from a linear regression model that also includes study center. For binary outcomes, intervention effect is the OR comparing intervention versus control groups, estimated from a logistic regression model that also includes study center. Only prespecified secondary analyses have regression results included; other results are given for context only. For HbA1c, although 147 participants had HbA1c measured at baseline (Table 1) and 249 at visit 4, only 63 participants had samples taken at both timepoints, antenatally and postnatally at 3 months, on account of sampling challenges during the COVID-19 pandemic. Results below show baseline and 3-month Hba1c data for participants tested at both timepoints. Median and IQR are shown in bold. GROW, gestation-related optimal weight centiles.

^a^In the control group: congenital hemangioma. In the intervention group: (1) 5-mm cyst in the perivascular space adjacent to the left lateral ventricle and (2) bilateral blepharoptosis.

There was no evidence of a significant difference in large-for-gestational-age (LGA) rates, neonatal intensive care unit (NICU) admission, estimated gestation age at birth or cord blood C-peptide concentrations between trial arms (Table 3).

### Safety outcomes

There were similar numbers of small-for-gestational-age (SGA) infants between trial arms, which fell within expected limits (Table 3). Rates of LGA, appropriate-for-gestational-age (AGA) and SGA infants were 18.4%, 77.9% and 3.7% for the control group and 20.3%, 74.5% and 5.2% for the intervention group respectively.

### Exploratory outcomes

An exploratory analysis was performed to assess the effects of weight loss: data were treated as a cohort and categorized into two groups according to weight loss or weight gain during the study. The proportion of participants who lost weight was not statistically different between the intervention and control arm (Supplementary Table 4).

Women who lost weight (154 of 389; 39.6%) had a higher BMI at enrollment (37.05 kg m^−^^2^ (6.29) versus 34.58 kg m^−^^2^ (6.22); *β* 2.19 kg (95% CI 0.93, 3.50; *P* = 0.001) and were more likely to be taking metformin (OR 2.25 (95% CI 1.16, 4.38); *P* = 0.017) at 36 weeks (Table 4). Women who lost weight had a mean weight change of −3.01 kg (3.60) from enrollment to 36 weeks gestation compared to +2.75 kg (2.74) in women who gained weight (Table 4).

**Table 4:** Effects of weight loss in pregnancy on maternal glycemia and pregnancy outcomes, with results summarized as mean and s.d. or median and IQR

	*n*	No weight loss *n* = 235	*n*	Weight loss *n* = 154	Regression coefficients and odds ratios (95% CI)	*P*
Maternal age (years)	235	32.62 (5.13)	154	33.64 (4.74)	1.07 (0.03, 2.10)	0.05
BMI (kg m^−^^2^)	254	34.58 (6.22)	154	37.05 (6.29)	2.19 (0.93, 3.46)	0.001
Weight at enrollment (kg)	234	92.28 (19.31)	154	99.85 (20.00)	6.68 (2.70, 10.66)	0.001
Self-reported ethnicity	235		154			
White		166 (70.64)		136 (88.31)		<0.001
Asian		57 (24.26)		11 (7.14)		
Black		10 (4.26)		6 (3.90)		
Other ethnic groups		2 (0.85)		1 (0.65)		
Primiparous	214	80 (37.38)	149	51 (34.23)	OR; 0.88 (0.56, 1.38)	0.58
Gestational weight gain pre-enrollment (kg)	234	3.97 (5.63)	154	3.88 (6.31)	−0.31 (−1.52, 0.91)	0.62
Maternal education (>degree)	235	114 (48.51)	154	77 (50.00)	OR; 1.10 (0.72, 1.68)	0.66
Index of multiple deprivation decile	228	6.63 (2.46)	150	6.70 (2.35)	0.21 (−0.27, 0.69)	0.39
Gestational diabetes in previous pregnancy	234	74 (31.62)	154	37 (24.03)	0.68 (0.42, 1.10)	0.12
Neonatal primary outcome						
Standardized birth weight (Intergrowth)	212	0.51 (0.99)	149	0.38 (0.89)	−0.16 (−0.37, 0.04)	0.11
		**0.54 (**−**0.06–1.17)**		**0.36 (**−**0.21–0.96)**		
Birth weight (g)	213	3,302.59 (471.37)	149	3,269.59 (447.63)	−41.47 (−140.38, 57.44)	0.41
Birth weight (Intergrowth centile)	212	64.59 (27.29)	149	60.91 (25.58)	−4.49 (−10.19, 1.22)	0.12
Neonatal secondary outcomes						
Large for gestational age (Intergrowth)	212	47 (22.17)	149	23 (15.44)	OR; 0.52 (0.29, 0.93)	0.027
Large for gestational age (GROW)	212	20 (9.39)	149	9 (6.04)	OR; 0.54 (0.23, 1.30)	0.16
NICU admission	213	21 (9.86)	149	13 (8.72)	OR; 0.80 (0.38, 1.73)	0.58
Estimated gestational age at birth (weeks)	214	38.4 (1.3)	149	38.6 (1.3)	0.20 (−0.07, 0.47)	0.15
Cord blood C-peptide (umol l^−^^1^)	52	291.7 (226.1)	45	247.6 (176.2)	−39.56 (−121.60, 42.48)	0.34
Maternal primary outcome						
Weight change (kg)	234	2.75 (2.74)	154	−3.01 (3.60)		
		**2.10 (1.10–3.60)**		**−1.90 (−3.30–1.00)**		
Weight at 36 weeks (kg)	234	95.14 (19.87)	154	96.85 (19.28)		
Maternal pregnancy outcomes						
Cesarean section	235	107 (45.53)	154	62 (40.26)	OR; 0.77 (0.50, 1.18)	0.23
Metformin at 36 weeks	184	41 (22.28)	121	45 (37.19)	OR; 2.25 (1.16, 4.38)	0.017
Short-acting insulin at 36 weeks	184	29 (15.76)	121	12 (9.92)	OR; 0.91 (0.36, 2.30)	0.84
Long-acting insulin at 36 weeks	184	69 (37.50)	122	35 (28.69)	OR; 0.82 (0.43, 1.58)	0.55
TIR (3.5–6.7 mmol l^−^^1^) at 36 weeks (%)	129	71.08 (19.27)	95	80.40 (15.76)	6.53 (2.06, 11.02)	0.004
		**76.79 (60.13–85.07)**		**84.92 (72.92–92.19)**		
TIR (3.5–7.8 mmol l^−^^1^) at 36 weeks (%)	129	87.36 (12.20)	95	93.20 (7.12)	4.13 (1.52 to 6.75)	0.002
		**91.05 (84.30**–**95.44)**		**95.83 (90.86**–**97.78)**		
CGM mean glucose at 36 weeks (mol l−^1^)	129	5.93 (0.86)	95	5.63 (0.68)	−0.22 (−0.41, −0.02)	0.028
CGM mean glucose at 36 weeks (mg dl−^1^)	129	106.89 (15.41)	95	101.46 (12.16)	−3.92 (−7.41, −0.43)	0.028
Systolic blood pressure (mmHg)	189	119.34 (13.49)	128	116.58 (12.61)	−2.87 (−5.49, −0.25)	0.032
Diastolic blood pressure (mmHg)	189	72.35 (10.34)	128	70.88 (10.05)	−1.44 (−3.54, 0.66)	0.18
Maternal postnatal outcomes at 3 months						
HbA1c (mmol mol^−1^)	132	37.16 (4.36)	105	36.30 (3.50)	−3.64 (−5.70, −1.57)	0.001
HbA1c (%)	132	5.55 (0.40)	105	5.47 (0.32)	−0.33 (−0.52, −0.14)	0.001
TIR (3.9–10.0 mmol l^−^^1^) (%)	103	96.74 (5.96)	85	97.92 (3.35)	0.92 (−0.67, 2.52)	0.26
		**98.95 (96.25–99.73)**		**98.86 (97.73–99.78)**		
CGM mean glucose (mmol l^−^^1^)	103	6.32 (0.81)	85	6.22 (0.66)	−0.06 (−2.78, 0.17)	0.63
CGM mean glucose (mg dl^−^^1^)	103	113.89 (14.62)	85	112.01 (11.81)	−1.00 (−5.01, 3.02)	0.63
Maternal weight (kg)	142	85.60 (19.07)	108	90.70 (17.76)	−3.34 (−4.85, −1.82)	<0.001
Maternal weight change from enrollment to 3 months postnatal	142	−4.42 (5.55)	108	−8.06 (6.02)	−3.35 (−4.86, −1.85)	<0.001
Maternal weight change from 36 weeks pregnancy to 3 months postnatal	142	−7.00 (5.31)	108	−5.22 (6.19)	1.81 (0.35, 3.27)	0.015
Maternal BMI (kg m^−^^2^)	142	32.22 (6.47)	108	33.41 (5.95)	−1.25 (−1.80, −0.70)	<0.001
Systolic blood pressure (mmHg)	132	118.96 (14.36)	104	118.45 (13.11)	0.56 (−2.72, 3.85)	0.74
Diastolic blood pressure (mmHg)	132	80.19 (13.05)	104	79.39 (12.96)	0.72 (−2.53, 3.97)	0.66
Safety outcomes						
Small for gestational age (Intergrowth)	212	11 (5.19)	149	4 (2.68)		
Stillbirth	235	0.00 (0.00)	154	0.00 (0.00)		
Neonatal death	235	0.00 (0.00)	154	0.00 (0.00)		
Maternal death	235	0.00 (0.00)	154	0.00 (0.00)		
Congenital anomaly^a^	213	1 (0.47)	149	1 (0.67)		

For continuous outcomes, effect measure is the baseline (where available)-adjusted difference in mean outcome between ʽNo weight lossʼ and ʽWeight lossʼ groups, estimated from a linear regression model that also includes study center. For binary outcomes, effect measure is the OR comparing ʽNo weight lossʼ and ʽWeight lossʼ groups, estimated from a logistic regression model that also includes study center. The number of subjects in this analysis (*n* = 389) is smaller than that given in Table 3. Participants could not be included if they had no data for weight at 36 weeks. Outcomes that ended the pregnancy before 36 weeks could not be included, such as stillbirth, neonatal death and maternal death. Median and IQR are shown in bold.

^a^In the no weight loss group: bilateral blepharoptosis. In the weight loss group: congenital hemangioma.

Weight loss was associated with significantly improved time in range (80.40% (15.76) versus 71.08% (19.27); *β* 6.53% (95% CI 2.06, 11.02); *P* = 0.004), continuous glucose monitoring (CGM) mean glucose (5.63 mmol l^−^^1^ (0.68) versus 5.94 mmol l^−^^1^ (0.86); *β* −0.22 mmol l^−^^1^ (95% CI −0.41, −0.02); *P* = 0.028); 101.46 mg dl^−^^1^ (12.16) versus 106.89 mg dl^−^^1^ (15.41); *β* −3.92 mg dl^−^^1^ (95% CI −7.41, −0.43); *P* = 0.028). Systolic blood pressure was also significantly reduced in women who lost weight (116.58 mmHg (12.61) compared to 119.34 mmHg (13.49); *β* −2.87 mmHg (95% CI −5.49, −0.25); *P* = 0.032) (Table 4).

Weight loss in late pregnancy was associated with reduced rates of LGA infants (OR 0.52 (95% CI 0.29, 0.93); *P* = 0.027; Table 4). Rates of LGA, AGA and SGA were 22.2%, 72.6% and 5.2% for the weight gain group and 15.4%, 81.9% and 2.7% for the weight loss group respectively. The number of SGA infants fell within expected limits in both groups. The effect of weight loss upon LGA appeared to be mediated through improved glycaemia at 36 weeks. When the model was adjusted for maternal time in range at 36 weeks, the association between LGA and weight loss was no longer significant (OR 0.90 (95% CI 0.41, 1.97); *P* = 0.787).

Weight loss in late pregnancy was maintained postnatally, associated with reduced weight (*β* −3.34 kg (95% CI −4.85, −1.82); *P* < 0.001) and BMI (*β* −1.25 kg m^−^^2^ (95% CI −1.80, −0.70); *P* < 0.001) at 3 months postpartum (Table 4). Weight loss in late pregnancy was associated with improved postnatal metabolic health including improved HbA1c (*β* −3.64 mmol mol^−^^1^ (95% CI −5.70, −1.57); *P* = 0.001; *β* −0.33% (−0.52, −0.14); *P* = 0.001).

**Extended Data Fig. 2:**
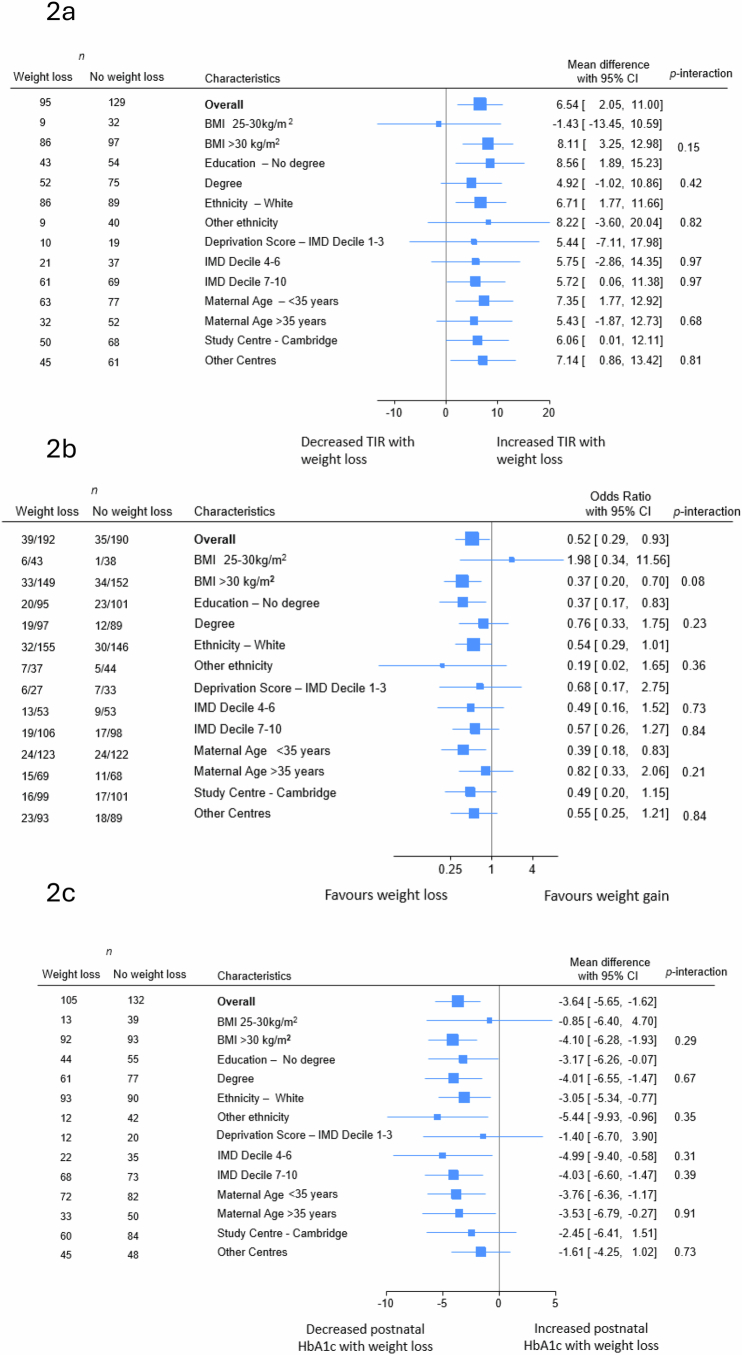
Post-hoc subgroup analyses to determine interaction effect of participant characteristics on the association between weight loss and pregnancy outcomes. Subgroup analyses were conducted for baseline BMI, education, ethnicity, socioeconomic status, maternal age, and study centre upon (**a**) Associations between weight loss upon CGM TIR (3.5–6.7 mmol/L) at 36 weeks. (**b**) Associations between weight loss and LGA (Intergrowth) in offspring. (**c**) Associations between weight loss and HbA1c (mmol/mol) at 3 months postnatally. Estimated effect sizes were calculated using unadjusted logistic regression for categorical outcomes and unadjusted linear regression for continuous outcomes. Effect sizes are shown as odds ratios or mean difference for each subgroup with 95% confidence interval. There were no significant interactions with the categories of maternal BMI, education, ethnicity, deprivation, age and study centre across any of the outcomes (*p*-interaction >0.05).

The association between weight loss and CGM time in range (TIR) (3.5–6.7 mmol l^−^^1^), LGA and postnatal HbA1c was not affected by maternal BMI at enrollment, education, ethnicity, deprivation score, maternal age or study center (Extended Data Fig. 2).

Participants taking metformin at 36 weeks gestation were more likely to lose weight (OR 2.01 (1.19–3.40; *P* = 0.009 after adjustment for trial arm and study center). However, results of the weight loss analysis were consistent in magnitude and direction even after additional adjustment for metformin use. After adjustment for metformin use at 36 weeks gestation in addition to study center and trial arm, participants in the weight loss group had evidence of reduced infant LGA (OR 0.48 (95% CI 0.24, 0.95); *P* = 0.034), reduced maternal systolic blood pressure (*β* −3.00 mmHg (95% CI −5.75, −0.25); *P* = 0.033), reduced maternal mean CGM glucose (in mg dl^−^^1^: *β* −3.94 mg dl^−^^1^ (95% CI −7.63–−0.25); *P* = 0.037; in mmol l^−^^1^: *β* −0.22 mmol l^−^^1^ (95% CI −0.42, −0.01; *P* = 0.037), increased maternal TIR at 36 weeks (*β* 6.22% (95% CI 1.47, 10.97); *P* = 0.011) and reduced postnatal HbA1c (in mmol mol^−^^1^: *β* −3.78 mmol mol^−^^1^ (95% CI −6.54, −1.010; *P* = 0.009).

**Extended Data Fig. 3:**
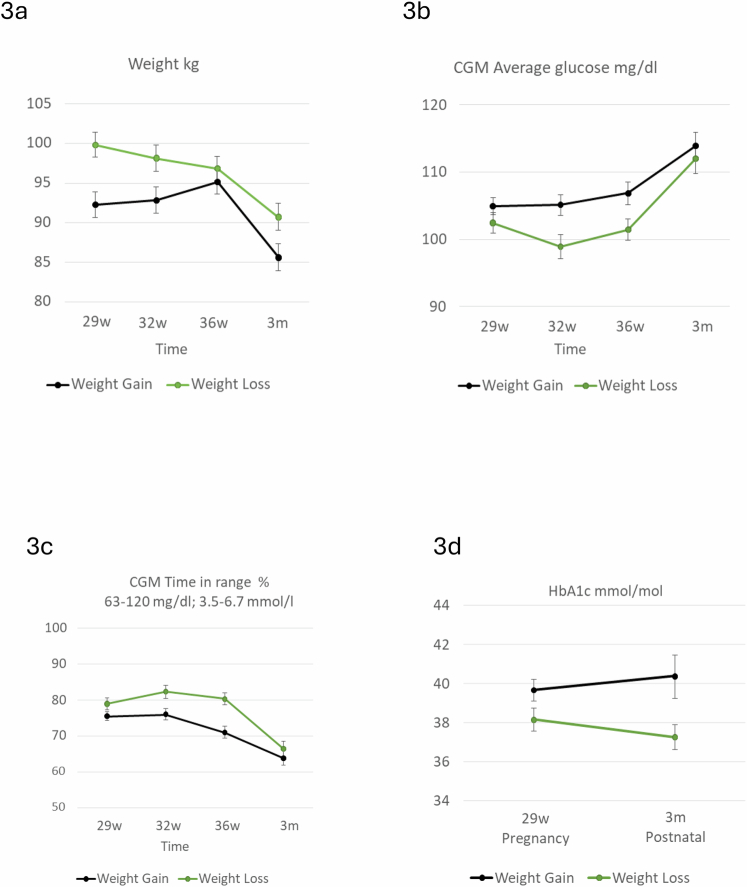
Changes in weight status and glycaemic control in women who lost weight versus gained weight from study enrolment to delivery. **(a)** Mean (SD) weight status at 29 weeks (n = 154;235), 32 weeks (n = 119;173), 36 weeks (n = 154;234) gestation, and 3 months postpartum (n = 108;142) is shown between women who lost weight versus gained weight, respectively. **(b)** Mean (SD) CGM average glucose (mg/dL) and **(c)** mean (SD) CGM Time in Range (63–120 mg/dL) at 29 weeks (n = 137;199), 32 weeks (n = 90;112), 36 weeks (n = 95;129) gestation, and 3 months postpartum (n = 85;103) is shown between women who lost weight versus gained weight, respectively. **(d)** Mean (SD) HbA1c (mmol/mol) at 29 weeks (n = 60;75) gestation and 3 months postpartum (n = 105;132) is shown between women who lost weight versus gained weight, respectively.

The timescales of changes in weight status, CGM TIR and average glucose, and postnatal HbA1c at each study timepoint between women who lost weight versus women who gained weight are shown in Extended Data Fig. 3.

### Sensitivity analyses

Adjustments for gestational age at birth, multiple imputation and maternal dietary adherence did not alter the neonatal primary outcome (Extended Data Tables 5–7).

### Post hoc analyses

As HbA1c at enrollment and postnatally was available only on a subset of participants due to COVID-19 restrictions, we assessed if this subset was representative of the larger cohort (Supplementary Table 5). Participants with an HbA1c at both timepoints were not statistically different to other participants in terms of maternal age, BMI, ethnicity, parity, baseline HbA1c (where available), oral glucose tolerance test (OGTT) results or baseline CGM metrics. However, they were more likely to have a degree (56% versus 46%), less likely to be a smoker (3% versus 12%), more likely to be diagnosed earlier (mean 20.5 versus 23.3 weeks) and more likely to be taking long-acting insulin at enrollment (40% versus 21%).

We compared groups that were categorized according to the presence of weight gain (>1 kg weight change), weight stability (±1 kg in weight change) and weight loss (>1 kg weight loss) from enrollment to 36 weeks gestation (Supplementary Table 6). Compared to the weight-stable group, weight loss was associated with improved glycaemia antenatally and postnatally, and a lower likelihood of LGA infants.

## Discussion

In women with gestational diabetes with a BMI ≥ 25 kg m^−^^2^, provision of an energy-restricted diet reduced the requirement for long-acting insulin. The difference in the energy content of the diets between the randomized groups was insufficient to bring about a significant difference in weight trajectories, and the primary maternal and neonatal outcomes did not differ. However, 40% of the whole cohort lost weight with no increase in adverse events. On secondary analysis, weight loss (an average of 3 kg or 3%) in late pregnancy was associated with improved maternal glycaemia antenatally and postnatally, reduced systolic blood pressure and reduced LGA infants. Modest weight loss in late pregnancy appeared safe in gestational diabetes.

Conventional dietary approaches to gestational diabetes, although burdensome for the patient, do not consistently offer improvements upon glycaemia and pregnancy outcomes. Multiple small studies have assessed different diets in gestational diabetes, usually through the provision of tailored dietary advice, but have had low statistical power to examine pregnancy outcomes. Although a reduced carbohydrate diet^12,13^ or reduced glycaemic index diet^4,14^ is widely recommended for clinical care in gestational diabetes, the evidence base for these recommendations is limited. For example, Yamamoto and colleagues^4^ used a meta-analysis to assess the effects of a low-carbohydrate diet (2018; three studies; total *n* = 194) or low-glycaemic-index diet (four studies; *n* = 304) on glycaemia and infant birth-weight outcomes. A low-glycaemic index diet was associated with 0.3 mmol l^−^^1^ (5.3 mg dl^−^^1^) and a 0.4 mmol l^−^^1^ (7.1 mg dl^−^^1^) reduction in fasting and postprandial glucose respectively (*n* = 195) while a low-carbohydrate diet showed no significant effect on fasting or postprandial glycaemia. Neither low-carbohydrate nor low-glycaemic-index diets alone improved birth weight or reduced medication requirements, but pooled analysis showed that any dietary intervention was associated with reduced medication requirements (15 studies; 1,023 patients) and a ~170 g reduction in birth weight but no effect on LGA infants (16 studies; 441 patients). Hernandez et al.^15^ examined a conventional lower-carbohydrate diet (40%) with higher fat (45%) against a complex-carbohydrate (60%) and lower-fat (25%) diet but did not see any difference in weight gain or TIR between these two diets^15^. Mijatovic et al.^16^ showed that a lower-carbohydrate diet in gestational diabetes did not improve average glucose concentration or HbA1c levels. Our data suggest that restricting energy content could provide a new dietary approach to gestational diabetes. However, our results suggest that modest weight loss is more likely to improve pregnancy outcomes compared to restricting gestational weight gain alone. Our results demonstrate that weight loss (an average of 3% or 3 kg) was associated with improved antenatal and postnatal glycaemia (0.3 mmol l^−^^1^ (5 mg dl^−^^1^)), improvement in mean CGM glucose, 7% improvement in TIR at 36 weeks (range 3.5–6.7 mmol l^−^^1^; 63–140 mg dl^−^^1^), reduced postnatal HbA1c and a reduction in LGA. As our study population received a low-glycaemic-index diet with 40% energy from carbohydrate, our results suggest that even with optimal dietary composition, energy restriction or weight loss provides additional benefits to mothers and infants. Our data is consistent with recent work by Johansson and colleagues^10^, which identified that reduced gestational weight gain, or even weight loss, could improve outcomes in pregnant women with obesity. Our study demonstrates that a reduced-energy diet was associated with a reduced requirement for long-acting insulin. This effect may be mediated directly by reduced energy intake itself or may be indirectly related to reduced portion size at dinner time, reduced insulin resistance or reduced carbohydrate intake. Future work will aim to clarify the main drivers of this effect.

Although our work demonstrated that a reduced-energy diet is safe and feasible in pregnancy, the optimal method for promoting weight loss in routine clinical care needs further exploration. While the diet boxes have been a successful and acceptable method of delivery of blinded research diets, provision of an energy-restricted diet alone was insufficient to promote significant weight loss in pregnancy, perhaps due to adherence or insufficient energy difference between arms. Tsirou and colleagues^17^ used a diet and exercise intervention but did not achieve weight loss (*n* = 43), with no resulting differences in pregnancy outcomes. Rae and colleagues^18^ provided personalized dietary advice but identified no benefits to a 30% energy restriction in 124 women with gestational diabetes. A more intensive approach was used by Magee et al.^19^, who admitted women for a week to promote weight loss. Their results demonstrated improvements in glycaemia and insulin resistance, but this is not feasible for widespread use. However, Hodson and colleagues^20^ delivered a successful weight reduction program in 14 pregnant women with gestational diabetes using dietary advice, which was feasible in a healthcare setting and well tolerated by participants.

Our work has several clinical implications. Energy restriction reduced insulin requirements and improved postnatal glycaemia, with further benefits on LGA rates for women who lost weight. These are important outcomes for patients and clinicians, which are not consistently improved by conventional dietary management of gestational diabetes. Weight loss (an average of 3 kg or 3%) in women with gestational diabetes was associated with improved antenatal glycaemia, with an improvement in TIR at 36 weeks of 7% and 4%, using ranges 3.5–6.7 mmol l^−^^1^ (63–120 mg dl^−^^1^) and 3.5–7.8 mmol l^−^^1^ (63–140 mg dl^−^^1^) respectively. The magnitude of this benefit cannot easily be compared to other studies, since few other interventions have demonstrated efficacy to improve CGM metrics in gestational diabetes^21^. Our data showing a 4 to 7% improvement in TIR are consistent with reports from other populations showing that a 5% improvement in TIR in pregnancy is clinically important^22^. Modest weight loss in late pregnancy was associated with a significant reduction in LGA infants, importantly with no increase in SGA infants. Prevention of LGA infants is likely to improve delivery outcomes but may also have life-long benefits, as LGA in infancy is associated with an increased risk of childhood obesity with accompanying increased cardiovascular and metabolic risk^23,24^. Previous work has yielded conflicting results regarding SGA infants. Xie and colleagues^9^ identified that women with gestational diabetes with gestational weight gain below the Institute of Medicine target ranges had higher rates of SGA infants but Wilkins and coworkers^11^ identified no increase in SGA infants in a similar cohort. SGA rates in infants in this cohort were within expected limits, regardless of trial arm or the presence of weight loss. The longer-term effects of weight loss in pregnancy upon child growth to 3 years of age will be assessed in the DiGest follow-up study^25^.

Preventing postnatal type 2 diabetes after gestational diabetes is crucial, particularly in view of the high risk of complications and early mortality in women with early onset type 2 diabetes^26^. Recent work has identified a 10-times increase in risk of type 2 diabetes in women with a history of gestational diabetes^27^. Our data demonstrate that interventions to address maternal weight in pregnancy may yield benefits upon postnatal HbA1c. In our study, a reduced-energy diet was associated with a reduction in postnatal HbA1c. Results of the exploratory analysis of weight loss suggest that further benefits upon postnatal HbA1c may be achieved by weight loss in pregnancy. Participants who lost 3 kg or 3% of weight in late pregnancy reduced postnatal HbA1c by 3.6 mmol mol^−^^1^ (0.33%). The magnitude of this effect is similar to that seen in people with type 2 diabetes, where there was a mean HbA1c reduction of 0.1% for each 1-kg weight loss^28^, suggesting that long-term weight loss in pregnancy is equally metabolically beneficial to postnatal weight loss, consistent with work by Lim and colleagues^29^. Although many women are motivated to lose weight postnatally, in practice the new demands of motherhood, sleeplessness, postnatal depression and reduced income make weight loss very challenging in the postnatal period. Our study demonstrates that weight loss in pregnancy is feasible and safe. It was maintained for at least 3 months postpartum. Longitudinal monitoring during the DiGest follow-up study (2022–2026) will identify if weight loss in pregnancy is sustained for up to 3 years postnatally, reducing rates of type 2 diabetes or prediabetes after gestational diabetes^25^.

A reduced-energy diet of around 1,200 kcal d^−1^ should be considered for evidence-based clinical practice internationally for women with gestational diabetes with a BMI ≥ 25 kg m^−^^2^. Our study was popular among women living with obesity, recruited from diverse socioeconomic groups, highlighting that an energy-restricted diet in pregnancy is acceptable and achievable to women. Future work should assess if the potential additional benefits of weight loss can be harnessed in a clinical setting, possibly supported by self-management and educational programs to promote additional benefits in the postnatal period. Most women with gestational diabetes with a BMI ≥ 25 kg m^−^^2^ will be able to safely follow an energy-restricted diet themselves, supported by the clinical diabetes in pregnancy care team but without additional medical supervision. Successful and healthy energy restriction in pregnancy could be achieved using a low-glycaemic-index diet, with plenty of vegetables, lean protein and some dairy products to ensure sufficient nutrients are included. Excluding whole food groups such as carbohydrates should be avoided as low-carbohydrate diets have not been shown to be safe in pregnancy^30^.

Our study assessed the effect of a reduced-energy diet in pregnancy in a diverse cohort of women recruited from eight study centers in the United Kingdom, with the collection of detailed data on maternal and neonatal outcomes. This study has several limitations. Our study population was more ethnically diverse than the UK population, but still had relatively small numbers of women from non-white backgrounds. Studies of dietary interventions typically use dietary advice as an intervention, preventing blinding of the participant or research team. We chose to use a whole-diet intervention to reduce bias, facilitate blinding and reduce socioeconomic, educational and cultural barriers to dietary adherence. The diet boxes also ensured that participants in both arms had access to adequate micronutrients for safety and allowed consistent macronutrient provision between arms. We relied on ordering information, food diaries and patient report to assess adherence, but did not ask for uneaten foods to be returned for quantification. However, the study team contacted the participants weekly via email or telephone to assess adherence and satisfaction, and compliance to the intervention was discussed at each study visit. If required, advice was provided by the study team to boost adherence, such as alternating different study meals to increase variety or splitting meals into two if they were struggling with portion size. The baseline BMI of our participants was higher than expected at 35.7 kg m^2^. Our control diet boxes included 2,000 kcal d^−1^, aligned to standard recommendations for energy requirements for women in the United Kingdom^31^, but this may have been an underestimate of energy needs to maintain weight stability in late pregnancy for this cohort with a relatively high mean BMI. We used double-blinding to reduce bias in the study, but this prevented opportunities for coaching women towards individual weight targets in the active treatment group. We used maternal weight change as our primary endpoint, but more sensitive measures of body composition may have yielded more information. While we recruited women as soon as possible after gestational diabetes diagnosis, most women received around 6 weeks of food in the diet boxes, which may have been too short a period for meaningful changes in primary outcomes. CGM metrics were used to assess maternal glycaemia antenatally and postnatally; results were masked to clinical and research teams. Restrictions upon face-to-face hospital attendance resulted in reduced sampling for HbA1c during the COVID-19 pandemic, affecting both baseline and postnatal results and thus limiting the sample size available for analysis. Subsequent analysis confirmed the statistical significance of the findings, but they should be interpreted cautiously. While these data are likely to be missing at random, women with an earlier diagnosis of gestational diabetes were more likely to have a baseline HbA1c, presumably because there was more time available for blood sampling to occur, for example, to coordinate with an antenatal face-to-face visit or scan. However, postpartum assessment of glycaemia included both HbA1c and CGM and will continue until 3 years postnatally in participants continuing in the follow-up study. Post hoc analysis exploring the interaction effect of different participant characteristics is also limited by the small sample size in some of the comparisons.

In conclusion, in women with gestational diabetes with a BMI ≥ 25 kg m^−^^2^, energy restriction to 1,200 kcal d^−1^ should be considered in evidence-based guidelines. Energy restriction in pregnancy was safe and reduced the requirement to start long-acting insulin in gestational diabetes.

## Methods

### Trial oversight

The DiGest trial was a randomized, controlled, double-blind, whole-diet intervention study with a parallel design conducted in eight hospital centers in England. The trial design and protocol were published previously^32^. The trial was funded by Diabetes UK and supported by a trial steering committee and data safety monitoring board (Supplementary Information). CGM equipment was supplied at reduced cost by Dexcom Inc. The funders had no influence on the design or conduct of the trial and were not involved in data collection or analysis, in the writing of the manuscript or in the decision to submit it for publication. The trial was conducted in accordance with the Declaration of Helsinki and the protocol was approved by the National Research Ethics Committee, United Kingdom (reference 18/WM/0191) and the NHS Health Research Authority (IRAS 242924; ISRCTN 65152174).

The COVID-19 pandemic necessitated several changes to the original protocol. The diagnostic criteria for gestational diabetes were expanded to include the Royal College of Obstetricians and Gynaecologists interim COVID-19 criteria during 2020–2022 (random glucose 9–11 mmol l^−^^1^ or HbA1c 41–47 mmol mol^−^^1^ at booking; fasting glucose ≥5.6 mmol l^−^^1^ or HbA1c ≥ 39 mmol mol^−^^1^ at 28 weeks gestation)^33^. Study visits were changed from hospital-based visits to home-based visits or virtual contacts. Baseline HbA1c results were limited because participants did not have this taken routinely during the pandemic. The postnatal OGTT at 6 weeks postpartum could not be performed and was replaced by CGM and HbA1c at 3 months postpartum. All changes were made in discussion with the trial sponsor, trial steering committee and National Research Ethics Committee.

### Study population

Women aged ≥18 years old with an ultrasound-confirmed singleton pregnancy, gestational diabetes diagnosed before 30 + 6 weeks gestation and a BMI ≥ 25 kg m^−^^2^ were recruited to the trial. The diagnosis of gestational diabetes was based on the criteria of the National Institute for Health and Care Excellence (75 g OGTT ≥ 5.6 mmol l^−^^1^ (≥100 mg dl^−^^1^) fasting and ≥7.8 mmol l^−^^1^ (≥140 mg dl^−^^1^) at 2 h; previous gestational diabetes, with glucometer testing recurrently above targets fasting ≥5.3 mmol l^−^^1^ fasting and ≥7.8 mmol l^−^^1^ 1 h after meal)^34^. Treatment of gestational diabetes in all centers followed the National Institute for Health and Care Excellence guidelines, offering a period of dietary change followed by metformin and/or insulin for women with persistent hyperglycemia^34^. Women were excluded if they had evidence of multiple pregnancy or severe congenital abnormality on ultrasound; had severe pre-existing comorbidities such as renal failure, liver disease, cardiac failure and psychiatric conditions requiring in-patient admission; were taking medications at the time of the OGTT that may have interfered with results (for example, high-dose oral steroids or immunosuppressants); had complications such as preterm labor, severe anemia or intrauterine growth restriction at gestational diabetes diagnosis; had HbA1c at diagnosis of gestational diabetes baseline of ≥48 mmol mol^−^^1^; had previously been diagnosed with diabetes; had specialized dietary requirements (for example, vegan or severe nut allergy); or had gestational weight loss of >5% comparing prepregnancy weight and weight at diagnosis.

### Trial procedures

The trial design is summarized in Extended Data Fig. 1. Patients were recruited, had a baseline visit and then were randomized to a trial arm and followed up at 32- and 36-weeks gestation and 12-weeks postpartum. Written informed consent was obtained from all participants. All data was entered into a database system Castor (v.2024.3.1.0).

### Randomization

The randomization protocol was designed in advance by one of the study statisticians (V.F.). The allocations were programmed into the food ordering website to ensure participants were automatically randomized while maintaining blinding. Randomization was implemented using the library ‘blockrand’ in the statistical package R. The randomization was done in permuted blocks of size 6, in a 1:1 ratio and stratified by center.

### Intervention and control

The diet boxes were developed in association with an industrial partner (Mayfield Foods Ltd). The diet boxes contain 2,000 kcal d^−1^ (control) or 1,200 kcal d^−1^ (reduced-energy intervention) comprising 40% carbohydrate, 25% protein and 35% fat. The menu range provided to the participants is shown in Supplementary Table 7.

### Outcomes

The DiGest trial has two coprimary endpoints: maternal weight change between enrollment and 36-weeks gestation, and neonatal birth weight, assessed using neonatal sex-appropriate s.d. scores (SDS), calculated for weight and length measurements (with adjustment for gestational age at birth) using customized centiles (Intergrowth and Grow UK 1990 growth reference using LMSgrowth software)^35,36^.

Secondary maternal outcomes include maternal weight, BMI, glycaemia (using CGM metrics as per the international TIR consensus recommendations)^21^, HbA1c, cardiometabolic health (blood pressure, lipids, fasting insulin, fasting glucose), maternal food choice and eating behavior, quality of life, treatments administered for gestational diabetes and birth modality/complications.

Secondary neonatal outcomes include gestational age at delivery, preterm delivery (<37 weeks), LGA or SGA, cord blood C-peptide, admission to the NICU, neonatal jaundice requiring phototherapy, Apgar scores, anthropometry, neonatal hypoglycemic (defined as a capillary glucose <2.6 mmol l^−^^1^ on one or more occasions within the first 48 h of life, starting at least 30 min after birth and necessitating treatment either with 40% glucose gel administered to the buccal mucosa and/or with intravenous dextrose), neonatal nasogastric feeding and feeding type on discharge from hospital. Infant feeding choices and feeding history will also be examined at 3 months postpartum.

### Statistical analysis

Maternal characteristics and study outcomes were described using mean (s.d.), median (IQR) and *n* (%) where appropriate. Primary and secondary outcomes used data sampled at a single timepoint only. In all analysis, participants with available data were included in the group to which they were randomized, regardless of their level of compliance. With stratification by study center, regression coefficients for linear or logistic regression, adjusted for baseline values for continuous outcomes, were used to assess intervention effects for all continuous or categorical outcomes respectively. The Missing Indicator Method was used to assess the potential impact of missing data on effect estimation^37^. Multiple imputation was used to investigate the impact of missing data on the intervention effect for the primary outcomes, assuming data were missing at random. Additional analysis was performed to assess the impact of gestational age at birth and adherence on the intervention effect. Safety analysis was performed to compare rates of SGA stillbirth, maternal death and neonatal death between groups, and are presented as *n* (%). Results were considered statistically significant when *P* < 0.05. This significance level was considered appropriate for two coprimary outcomes because each outcome is tested independently in separate populations, maintaining the overall type I error rate for each population. Analysis was performed in STATA (v.17.0; StataCorp).

In an exploratory post hoc analysis recommended by the Trial Steering Committee, regression models were also used to compare outcomes between participants that lost weight and those who gained weight, irrespective of intervention assignment. To maintain consistency with the analysis plan, comparison of participants who lost weight with those who gained weight was performed using linear or logistic regression, with clustering for study center and adjustment for trial arm and (for continuous variables) the baseline measurement of the variable.

Post hoc subgroup analysis was also performed to assess the interaction of intervention effect of maternal BMI at enrollment, education, ethnicity, deprivation score, maternal age and study center on requirement for long-acting insulin. These interactions were also analyzed for the effect of weight loss on CGM TIR (3.5–6.7 mmol l^−^^1^) at 36 weeks, LGA and postnatal HbA1c.

### Sample size calculation

The original sample size was *n* = 500, which provided >90% power to identify a 0.33 s.d. (1 kg) difference in maternal weight change between groups (maternal primary outcome) and >90% power for identification of a 0.3 s.d. (150 g) difference in standardized birth weight (neonatal primary outcome), allowing for 20% withdrawals, with a significance level of 5% for each of the two primary outcomes (two-sided). However, in May 2022, the data safety monitoring board recommended reducing the sample size to 380 following an interim analysis after 250 participants were recruited. Using the data collected to that stage, the probability of finding the original effect size was calculated to be 0.72 if 380 women were recruited and 0.85 for both outcomes if 500 women were recruited. The data safety monitoring board therefore considered that the trial should not be stopped for futility after *n* = 250, but that 380 participants was sufficient to identify if significant differences were present. These recommendations were peer reviewed prior to implementation. We monitored withdrawal rates during the trial prior to the collection of primary endpoint data (11%) and therefore recruited 428 women to ensure there was primary outcome information available for 380 pregnancies.

### Ethics and inclusion

The protocol was approved by the National Research Ethics Committee, United Kingdom (reference 18/WM/0191) and the NHS Health Research Authority (IRAS 242924; ISRCTN 65152174). We recruited participants to this study regardless of age, gender, religion, ethnicity or political views. This study included pregnant individuals who were assigned female sex at birth. We did not exclude pregnant people based upon gender at the time of recruitment. For infants, we collected information on neonatal sex from medical records.

### Reporting summary

Further information on the research design is available in the Nature Portfolio Reporting Summary linked to this article.

## Online content

Any methods, additional references, Nature Research reporting summaries, source data, extended data, supplementary information, acknowledgements, peer review information; details of author contributions and competing interests; and statements of data and code availability are available at 10.1038/s41591-024-03356-1.

## Supplementary information

Supplementary Information Supplementary Tables 1–7, List of Steering Committee Members, List of Data Monitoring and Safety Board Members, List of Investigators (listed in alphabetical order by institution), List of Research Teams, Inclusion Criteria, Exclusion Criteria, Definitions of Trial Outcomes.

Reporting Summary

## Figures and Tables

**Extended Data Table 1:**
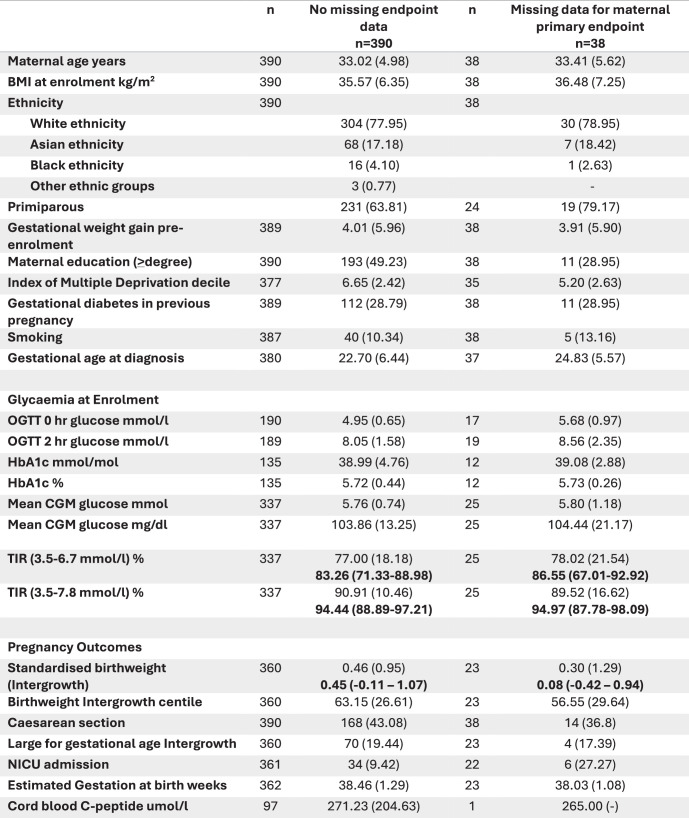
Characteristics of participants with and without missing data for the primary maternal outcome summarising key baseline characteristics separately in those with and without missing data for each primary outcome

Results are presented as mean (SD) or n (%) or median (IQR) in bold as appropriate. CGM: continuous glucose monitoring; NICU: Neonatal intensive care unit; OGTT: oral glucose tolerance test; TIR: time in range.

**Extended Data Table 2:**
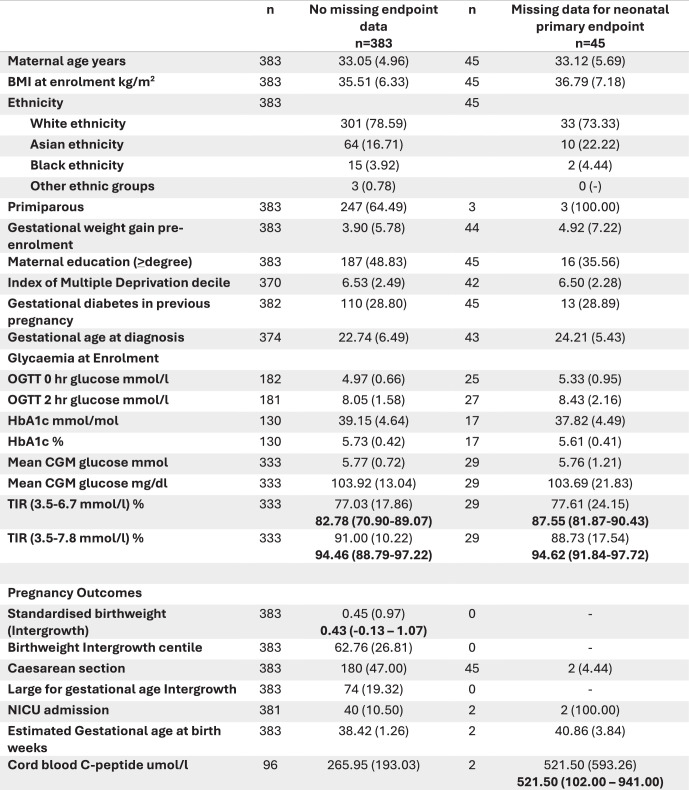
Characteristics of participants with and without missing data for primary neonatal outcome

Results are presented as mean (SD) or n (%) or median (IQR) in bold as appropriate. CGM: continuous glucose monitoring; NICU: Neonatal intensive care unit; OGTT: oral glucose tolerance test; TIR: time in range.

**Extended Data Table 3:**
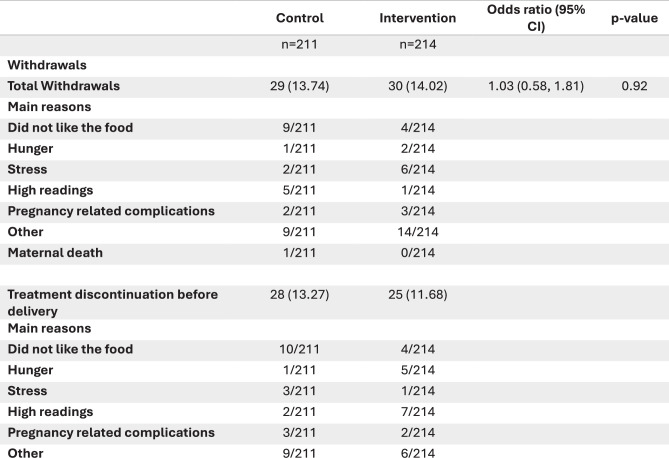
Withdrawals

13.8% (59/425) withdrew from the study, lower than the 20% expected *a priori*. Results are summarised as mean and SD. Differences between intervention and control groups are reported as odds ratios. Regression models are adjusted for study centre.

**Extended Data Table 4:**
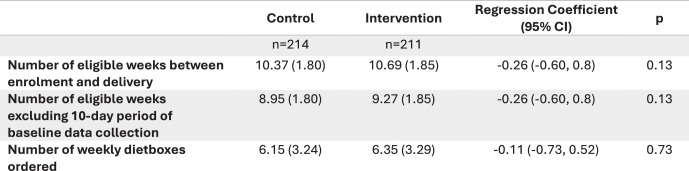
Measurement of adherence of the intervention/ control dietboxes

Results are summarised as mean and SD. Differences between intervention and control groups are reported as regression coefficients. Regression models are adjusted for study centre and the gestational age at birth.

**Extended Data Table 5:**
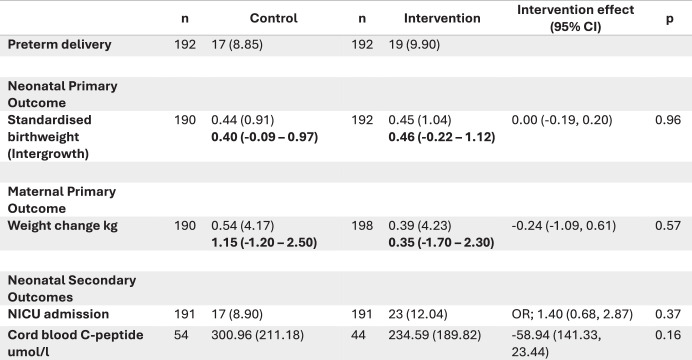
Sensitivity analysis 1: (a sensitivity analysis for the primary outcomes was performed including women with early deliveries, with adjustment for gestational age at delivery)

Results are summarised as mean and SD or as median IQR (bold font). Differences between intervention and control groups are reported as beta-coefficients (95% CI; continuous outcomes) or odds ratios (OR; binary outcomes). Regression models are adjusted for study centre, the baseline variable, and gestational age at birth (weeks).

NICU: Neonatal intensive care unit.

**Extended Data Table 6:**
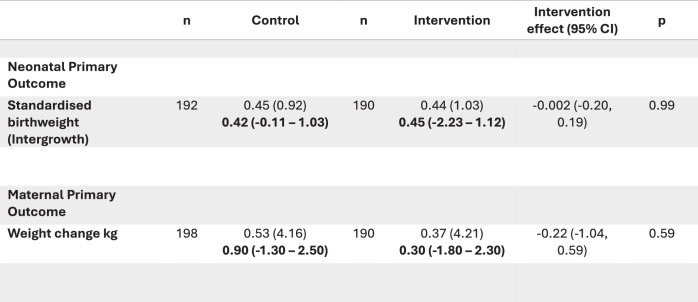
Sensitivity analysis 2: (including all recruited participants using multiple imputation

Results are summarised as mean and SD or median IQR (bold font). Differences between intervention and control groups are reported as beta-coefficients (95% Confidence Interval). Regression models are adjusted for study centre and the baseline variable.

**Extended Data Table 7:**
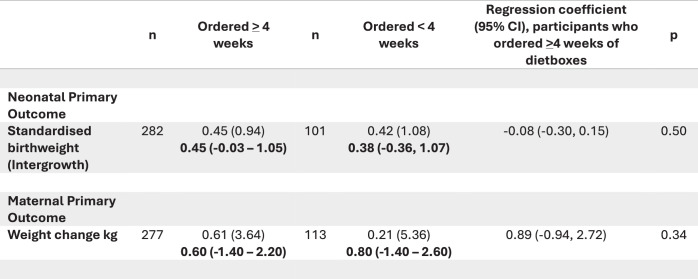
Sensitivity analysis 3: Effect of adherence to > = 4wks

Results are summarised as mean and SD or median IQR (bold font). Differences between participants who ordered over 4 weeks and those who ordered less than 4 weeks are reported as regression coefficients (95% Confidence Interval). Regression models are adjusted for study centre and the baseline variable.

**Justification: 115/392 (29.3%) in trial at 36 weeks ordered less than 4 weeks of food.

## Data Availability

To adhere to General Data Protection Regulation (https://gdpr-info.eu/), data will not be uploaded to a repository in advance of publication due to the potential for subject identification. Anonymized individual participant data is available upon request from the corresponding author (cm881@leicester.ac.uk), subject to approval from trial steering groups and data sharing and processing agreements. The timeframe for responding to data requests from the authors is within 1 month.

## Code availability

Stata code is available for this trial upon request from the corresponding author.
