# Supplementary material for: Reduced-energy diet in women with gestational diabetes: the dietary intervention in gestational diabetes DiGest randomized clinical trial
Source: Nat Med. 2025 Feb 19;31(2):514–23. doi: 10.1038/s41591-024-03356-1 (PMC11839452; doi:10.1038/s41591-024-03356-1)
Supplement: Supplementary file 1 — Supplementary Tables 1–7, List of Steering Committee Members, List of Data Monitoring and Safety Board Members, List of Investigators (listed in alphabetical order by institution), List of Research Teams, Inclusion Criteria, Exclusion Criteria, Definitions of Trial Outcomes. [file 41591_2024_3356_MOESM1_ESM.pdf]

nature medicine

Supplementary information https://doi.org/10.1038/s41591-024-03356-1

Reduced-energy diet in women with gestational diabetes: the dietary intervention in gestational diabetes DiGest randomized clinical trial

In the format provided by the authors and unedited

Supplementary Material

Supplementary Appendix

Supplementary Figures

Supplementary Tables

List of Steering Committee Members

List of Data Monitoring & Safety Board Members

List of Investigators (Listed in alphabetical order by institution.)

List of Research Team Members

Inclusion Criteria

Exclusion Criteria

Definitions of Trial Outcomes

Supplementary Figure 1: The design of the DiGest randomized controlled trial.

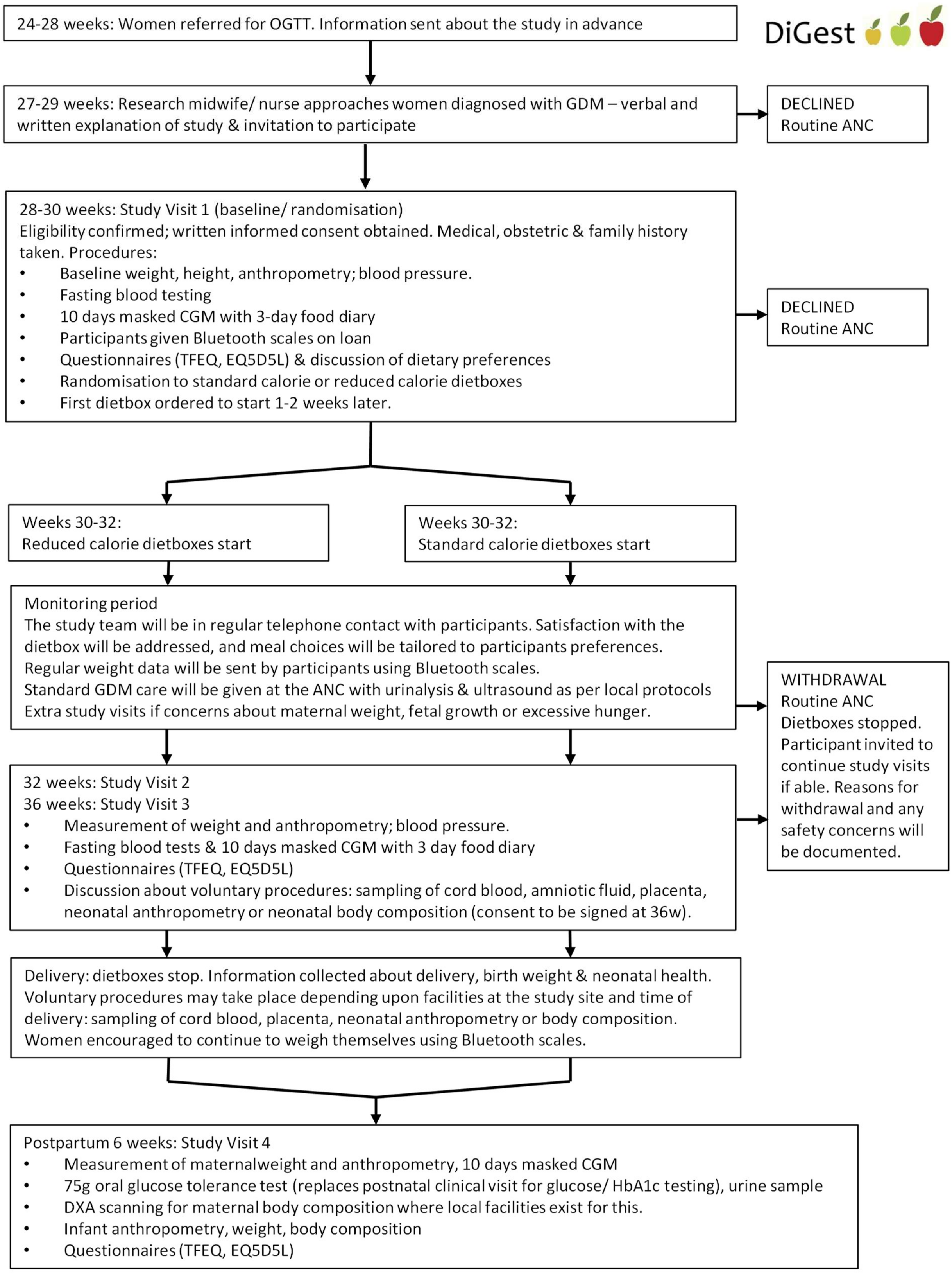

Supplementary Table 1: Participant satisfaction with dietboxes in control and intervention groups. Results are summarised as n (%). Differences between intervention and control groups are reported as beta-coefficients (continuous outcomes). Regression models are adjusted for study centre. Data were collected by the food company (Mayfield) using an anonymous online questionnaire at Survey Monkey.

	Control	Intervention	Differences: Beta Coefficient (95% CI)	p
	n=214	n=211		
**Completed satisfaction questionnaire on ≥1 occasion**	68 (31.78%)	74 (35.07%)	-0.02 (-0.12, 0.08)	0.739
**Completed satisfaction questionnaire from women who withdrew**	30 (14.02%)	29 (13.74%)	0.02 (-0.19, 0.23)	0.838
**Overall satisfaction score for quality of food**				
**% Highly satisfied**	28 (41.18%)	27 (36.49%)		
**% Satisfied**	30 (44.12%)	33 (44.59 %)		
**% Neither satisfied nor dissatisfied**	6 (8.82%)	8 (10.81%)		
**% Dissatisfied**	4 (5.88%)	5 (6.76%)		
**% Very dissatisfied**	0 (0%)	1 (1.35%)		
**Overall satisfaction score for quality of service**				
**% Highly satisfied**	43 (65.15%)	43 (59.72%)		
**% Satisfied**	17 (25.76%)	26 (36.11%)		
**% Neither satisfied nor dissatisfied**	4 (6.06%)	3 (4.17%)		
**% Dissatisfied**	1 (1.52%)	0 (0%)		
**% Very dissatisfied**	1 (1.52%)	0 (0%)		

Supplementary table 2: Additional secondary outcomes, to fulfil requirements for the core outcomes set for diabetes in pregnancy

Core Outcomes	All Participants	Control	Intervention
**Pre-eclampsia**	**7/383**	**2/192**	**5/191**
**Hypertensive disorders in pregnancy**	**19/383**	**8/192**	**11/191**

Supplementary table 3: Effect of intervention upon maternal health-related quality of life (HR-QOL).

	Control	Intervention	Intervention effect (95% CI)	p
**HR-QOL AT ENROLMENT**	n=209	n=209		
**Mobility**	1.48 (0.71)	1.46 (0.69)		
**Self-care**	1.13 (0.40)	1.11 (0.36)		
**Usual activity**	1.47 (0.69)	1.50 (0.68)		
**Pain**	1.89 (0.84)	1.85 (0.74)		
**Anxiety/depression**	1.67 (0.87)	1.60 (0.77)		
**Overall (HR-QOL index)**	0.78 (0.19)	0.79 (0.16)		
**HR-QOL AT 36 WEEKS**	n=144	n=143		
**Mobility**	1.83 (0.91)	1.66 (0.83)	-0.08 (-0.26, 0.09)	0.34
**Self-care**	1.27 (0.56)	1.22 (0.50)	-0.01 (-0.13, 0.11)	0.88
**Usual activity**	1.84 (0.87)	1.66 (0.76)	-0.16 (-0.33, 0.003)	0.06
**Pain**	2.18 (0.81)	2.07 (0.76)	0.05 (-0.22, 0.11)	0.51
**Anxiety/depression**	1.56 (0.78)	1.50 (0.70)	-0.09 (-0.24, 0.06)	0.23
**Overall (HR-QOL index)**	0.72 (0.20)	0.75 (0.18)	0.02 (-0.01, 0.06)	0.25
**HR-QOL AT 3 MONTHS POSTPARTUM**	n=130	n=138		
**Mobility**	1.19 (0.47)	1.12 (0.39)	-0.06 (-0.16, 0.04)	0.26
**Self-care**	1.08 (0.39)	1.04 (0.25)	-0.03 (-0.11, 0.05)	0.44
**Usual activity**	1.19 (0.43)	1.15 (0.47)	-0.02 (-0.12, 0.09)	0.71
**Pain**	1.50 (0.73)	1.47 (0.71)	0.02 (-0.15, 0.18)	0.84
**Anxiety/depression**	1.61 (0.87)	1.48 (0.74)	-0.11 (-0.29, 0.06)	0.21
**Overall (HR-QOL index)**	0.86 (0.16)	0.88 (0.14)	0.01 (-0.03, 0.04)	0.69

Supplementary table 4: Number of participants who experienced weight loss or weight gain in the intervention and control arms

	Intervention	Control
**Weight loss**	85/214 (39.7%)	69/211 (32.7%)
**Weight gain**	113/214 (52.8%)	121/211 (57.3%)

Supplementary table 5: Baseline characteristics in women with HbA1c data available at both antenatal and postnatal timepoints in comparison to those with missing data. Data are represented as mean (SD) or n (%). Data also presented as median (IQR) are shown in bold.

	**n**	**All Participants n=425**	**n**	**Participants with HbA1c at two timepoints n=63**	**n**	**Participants with missing data for HbA1c at one or more timepoints n=365**	**p**
**Maternal age years**	425	33.03 (5.04)	63	33.64 (4.57)	365	32.96 (5.11)	0.26
**BMI kg/m^2**	425	35.67 (6.44)	63	36.20 (6.01)	365	35.55 (6.51)	0.83
**Self-reported ethnicity**	425		63		365		0.42
**White**		332 (78.12)		50 (79.37)		284 (77.81)	
**Asian**		73 (17.18)		8 (12.70)		66 (18.08)	
**Black**		17 (4.00)		4 (6.35)		13 (3.56)	
**Other ethnic groups**		3 (0.71)		1 (1.59)		2 (0.55)	
**Primiparous**	385	224 (52.68)	62	37 (59.68)	324	213 (65.74)	0.62
**Gestational weight gain pre-enrolment kg**	424	3.94 (5.89)	63	3.88 (5.86)	364	4.03 (5.97)	0.44
**Maternal education (≥degree)**	425	201 (47.29)	63	35 (55.56)	364	168 (46.03)	0.02
**Index of Multiple Deprivation decile**	412	6.53 (2.47)	63	6.73 (2.60)	349	6.49 (2.44)	0.12
**Gestational diabetes in previous pregnancy**	424	122 (28.77)	63	16 (25.40)	364	107 (29.40)	0.50
**Health at Enrolment**							
**Smoking**	422	44 (10.43)	61	2 (3.28)	364	43 (11.81)	0.017
**Physical activity PAEE (kJ/kg/d)**	230	19.86 (12.65)	34	18.96 (14.79)	196	20.03 (12.28)	0.86
**Habitual energy intake kcal/day**	215	1567.75 (653.40)	34	1658.60 (725.60)	181	1550.69 (639.68)	0.31
**Basal Metabolic Rate J/(h/kg)**	385	1643.07 (227.58)	62	1668.30 (214.40)	324	1637.56 (224.87)	0.66
**Systolic blood pressure mmHg**	418	115.69 (12.47)	59	112.97 (12.33)	362	116.13 (12.43)	0.27
**Diastolic blood pressure mmHg**	418	69.29 (10.12)	59	67.88 (10.79)	362	69.51 (9.96)	0.72
**Diagnosis**							
**Gestational age at diagnosis**	414	22.85 (6.40)	63	20.47 (8.37)	354	23.32 (5.89)	0.006
**OGTT 0 hr glucose mmol/l**	206	5.01 (0.71)	17	5.11 (0.90)	190	5.00 (0.69)	0.18
**OGTT 2 hr glucose mmol/l**	207	8.11 (1.67)	16	8.46 (2.04)	192	8.07 (1.63)	0.54
**HbA1c mmol/mol**	147	39.00 (4.63)	63	38.78 (5.26)	84	39.17 (4.11)	0.42
**HbA1c %**	147	5.72 (0.42)	63	5.70 (0.48)	84	5.74 (0.38)	0.42
**Medication Use at Enrolment**							
**Metformin**	425	94 (22.12)	63	16 (25.40)	365	79 (21.64)	0.89
**Short-acting insulin**	425	38 (8.94)	63	9 (14.29)	365	29 (7.95)	0.55

**Long-acting insulin**	425	101 (23.76)	63	25 (39.68)	365	76 (20.82)	0.037
	**n**	**All Participants n=425**	**n**	**Participants with HbA1c at two timepoints n=63**	**n**	**Participants with missing data for HbA1c at one or more timepoints n=365**	
**GLYCAEMIA AT ENROLMENT**							
**Days of CGM use**	361	5.79 (2.24)	54	6.10 (2.17)	308	5.73 (2.25)	0.22
**Mean CGM glucose mmol/l**	361	5.77 (0.77)	54	5.70 (0.76)	308	5.78 (0.77)	0.45
**Mean CGM glucose mg/dl**	361	103.95 (13.89)		102.77 (13.73)	308	104.10 (13.94)	0.45
**TIR (3.5-6.7 mmol/l) %**	361	77.02 (18.40) **83.30 (70.95 – 89.16)**	54	77.92 (18.85) **85.09 (75.98-89.16)**	308	76.92 (18.35) **83.22 (70.79-89.37)**	0.56
**TAR (3.5-6.7 mmol/l) %**	361	21.32 (19.18) **15.05 (7.87-28.61)**	54	20.33 (19.46) **12.90 (8.33-22.19)**	308	21.43 (19.16) **15.28 (7.83-28.78)**	0.56
**TBR (3.5-6.7 mmol/l) %**	361	1.66 (2.91) **0.53 (0.00-1.81)**	54	1.75 (2.01) **0.84 (0.17-3.07)**	308	1.65 (3.03) **0.49 (0.00-1.71)**	0.82
**TIR (3.5-7.8 mmol/l) %**	361	90.80 (10.98) **94.46 (88.79 – 97.31)**	54	90.72 (11.72) **94.21 (90.37-97.26)**	308	90.83 (10.85) **94.51 (88.36-97.31)**	0.81
**TAR (3.5-7.8 mmol/l) %**	361	7.54 (11.30) **3.20 (1.17-8.91)**	54	7.53 (12.06) **3.01 (1.04-6.56)**	308	7.52 (11.18) **3.23 (1.18-8.95)**	0.77
**TBR (3.5-7.8 mmol/l) %**	361	1.66 (2.91) **0.53 (0.00-1.81)**	54	1.75 (2.01) **0.84 (0.17-3.07)**	308	1.65 (3.03) **0.49 (0.00-1.71)**	0.82
**CV**	361	18.22 (3.84)	54	18.48 (4.28)	308	18.16 (3.76)	0.89
**SD**	361	1.05 (0.29)	54	1.06 (0.34)	308	1.05 (0.28)	0.89

42

Supplementary Table 6: Comparison of weight gain, weight loss and weight stable groups

The safety of weight loss in pregnancy: associations between weight loss, weight stability and weight gain upon maternal glycaemia and pregnancy outcomes. Weight loss was defined as weight loss of >1kg from enrolment to 36 weeks' gestation. Weight gain was defined as weight gain of >1kg from enrolment to 36 weeks' gestation. The weight stable group was defined as weight loss of < +/- 1kg from enrolment to 36 weeks' gestation. Results are summarised as mean and SD or median IQR (bold font). For continuous outcomes, intervention effect is the baseline (where available)-adjusted difference in mean outcome between intervention and control groups, estimated from a linear regression model that also includes study centre. For binary outcomes, intervention effect is the odds ratio comparing intervention vs control groups, estimated from a logistic regression model that also includes study centre. P-values <0.05 are considered statistically significant and the confidence intervals are two-sided. CGM: continuous glucose monitoring; GROW: gestation-related optimal weight centiles; NICU: neonatal intensive care unit; TIR: time in range. The number of subjects in this analysis (n=390) is smaller than that given in table 3. Participants could not be included if they had no weight data at 36 weeks' gestation. Outcomes which ended the pregnancy before 36 weeks could not be included, such as stillbirth, neonatal death and maternal death

	n	Weight stable n=92	n	Weight Loss n=119	n	Weight gain n=179	Differences Weight loss vs weight stable Ref: Weight stable	p	Differences Weight gain vs weight stable Ref: Weight stable	p	Differences Weight gain vs Weight loss Ref: Weight gain	p
**Maternal age years**	92	33.07 (4.02)	119	33.86 (4.99)	179	32.45 (5.35)	0.78 (-0.59, 2.14)	0.27	-0.76 (-2.03, 0.51)	0.24	1.54 (0.35, 2.72)	0.011
**BMI kg/m^2**	92	34.65 (5.59)	119	37.50 (6.40)	179	34.75 (6.43)	2.83 (1.15, 4.50)	0.001	0.56 (-0.99, 2.11)	0.48	2.27 (0.82, 3.71)	0.002
**Weight at enrolment kg**	92	92.28 (16.51)	119	101.41 (20.74)	179	92.89 (20.07)	8.99 (3.73, 14.24)	0.001	2.02 (-2.85, 6.89)	0.42	6.97 (2.43, 11.52)	0.003
**Self-reported ethnicity**	92		119		179			0.33		0.034		<0.001
**White**		74 (80.43)		103 (86.55)		127 (70.95)						
**Asian**		13 (14.13)		9 (7.56)		45 (25.14)						
**Black**		3 (3.26)		6 (5.04)		7 (3.91)						
**Other ethnic groups**		2 (2.17)		1 (0.84)		0 (0.00)						
**Primiparous**	90	29 (32.22)	114	74 (64.91)	158	62 (39.24)	OR; 1.18 (0.65, 2.15)	0.58	OR; 1.45 (0.83, 2.54)	0.19	OR; 0.81 (0.49, 1.36)	0.43

**Gestational weight gain pre-enrolment kg**	92	3.82 (4.74)	119	3.83 (6.77)	178	4.24 (5.96)	-0.18 (-1.81, 1.44)	0.83	0.50 (-1.01, 2.00)	0.52	-0.68 (-2.08, 0.73)	0.34
**Maternal education (≥degree)**	92	48 (52.17)	119	59 (49.58)	179	85 (47.49)	OR; 0.87 (0.50, 1.53)	0.64	OR; 0.73 (0.43, 1.22)	0.23	OR; 1.20 (0.74, 1.96)	0.45
**Index of Multiple Deprivation decile**	88	6.46 (2.57)	116	6.72 (2.39)	173	6.71 (2.37)	0.27 (-0.37, 0.91)	0.41	-0.03 (-0.62, 0.57)	0.93	0.30 (-0.25, 0.84)	0.29
**Gestational diabetes in previous pregnancy**	91	31 (34.07)	119	27 (22.69)	179	54 (30.17)	OR; 0.54 (0.29, 1.02)	0.06	OR; 0.77 (0.44, 1.34)	0.36	0.70 (0.40, 1.23)	0.21
**Neonatal Primary Outcome**												
**Standardised birthweight (Intergrowth)**	89	0.39 (0.84) 0.37 (-0.09 – 1.03)	114	0.40 (0.91) 0.37 (-0.17 – 0.97)	157	0.54 (1.03) 0.64 (-0.05 – 1.21)	-0.02 (-0.29, 0.24)	0.87	0.17 (-0.08, 0.42)	0.18	-0.20 (-0.43, 0.04)	0.10
**Birthweight g**	89	3228.76 (461.08)	114	3288.53 (446.84)	158	3325.23 (471.89)	51.08 (-78.69, 180.84)	0.44	100.18 (-21.32, 221.467	0.11	-49.10 (-163.10, 64.90)	0.40
**Birthweight Intergrowth centile**	89	61.80 (25.48)	114	61.26 (25.69)	157	65.29 (27.88)	-1.22 (-8.68, -6.24)	0.75	4.07 (-2.93, 11.07)	0.25	-5.29 (-11.85, 1.28)	0.11
**Neonatal Secondary Outcomes**												
**Large for gestational age Intergrowth**	89	15 (16.85)	114	17 (14.91)	157	38 (24.20)	OR; 0.71 (0.32, 1.56)	0.40	OR; 1.69 (0.85, 3.34)	0.13	OR; 0.42 (0.21, 0.83)	0.012
**Large for gestational age GROW**	89	6 (6.74)	114	7 (6.14)	158	16 (10.13)	OR; 0.84 (0.27, 2.67)	0.77	OR; 1.74 (0.64, 4.72)	0.28	OR; 0.49 (0.19, 1.27)	0.14
**NICU admission**	88	11 (12.50)	114	9 (7.89)	159	14 (8.81)	OR; 0.52 (0.20, 1.38)	0.19	OR; 0.66 (0.28, 1.55)	0.34	OR; 0.79 (0.31, 2.00)	0.62

**Estimated Gestational age at birth weeks**	89	38.31 (1.45)	114	38.62 (1.30)	159	38.43 (1.19)	0.35 (-0.003, 0.71))	0.05	0.09 (-0.24, 0.43)	0.58	0.26 (-0.05, 0.58)	0.10
**Cord blood C-peptide umol/l**	22	271.27 (188.63)	34	256.77 (193.28)	41	283.20 (225.09)	-14.36 -126.49, 97.76)	0.80	1.35 (-105.30, 107.99)	0.98	-15.71 (-110.98, 79.55)	0.74
**Maternal Primary Outcome**												
**Weight change kg**	92	0.08 (0.54)	119	-3.74 (3.79)	179	3.51 (2.79)	-3.74 (-4.49, -2.98)	<0.001	3.45 (2.75, 4.15)	<0.001	-7.19 (-7.85, -6.54)	<0.001
**Weight at 36 weeks kg**	92	92.36 (16.39)	119	97.70 (20.04)	179	96.40 (20.78)	-3.65 (-4.42, -2.89)	<0.001	3.48 (2.78, 4.17)	<0.001	-7.13 (-7.79, -6.47)	<0.001
**Maternal Pregnancy Outcomes**												
**Caesarean section**	92	40 (43.48)	119	47 (39.50)	179	81 (45.25)	OR; 0.82 (0.47, 1.44)	0.49	OR; 1.15 (0.69, 1.93)	0.59	OR; 0.71 (0.44, 1.16)	0.17
**Metformin at 36 weeks**	72	20 (27.78)	95	37 (38.95)	138	29 (21.01)	OR; 1.10 (0.48, 2.50)	0.82	OR; 0.51 (0.22, 1.18)	0.12	OR; 2.14 (1.00, 4.58)	0.05
**Short-acting insulin at 36 weeks**	72	12 (16.67)	95	9 (9.47)	138	20 (14.49)	OR; 0.36 (0.12, 1.13)	0.08	OR; 0.29 (0.10, 0.89)	0.030	OR; 1.25 (0.38, 4.05)	0.71
**Long-acting insulin at 36 weeks**	73	24 (32.88)	95	29 (30.53)	138	51 (36.96)	OR; 0.99 (0.42, 2.34)	0.99	OR; 1.11 (0.50, 2.50)	0.79	OR; 0.89 (0.43, 1.85)	0.75
**TIR (3.5-6.7 mmol/l) at 36 weeks %**	55	76.15 (19.39)	77	79.52 (16.19)	92	70.56 (18.73)	3.66 (-2.13, 9.44)	0.21	-2.57 (-8.23, 3.09)	0.37	6.23 (1.09, 11.36)	0.018
**TIR (3.5-7.8 mmol/l) at 36 weeks %**	55	90.75 (10.01)	77	92. 95 (7.38)	92	86.69 (12.59)	1.98 (-1.36, 5.32)	0.24	-2.75 (-6.01, 0.51)	0.10	4.73 (1.77, 7.69)	0.002
**CGM mean glucose at 36 weeks mmol/l**	55	5.77 (0.81)	77	5.66 (0.69)	92	5.95 (0.85)	-0.16 (-0.41, 0.09)	0.21	0.02 (-0.23, 0.26)	0.88	-0.18 (-0.41, 0.04)	0.10

**CGM mean glucose at 36 weeks mg/dL**	55	103.92 (14.59)	77	101.94 (12.47)	92	107.21 (15.34)	-2.90 (- 7.43, 1.63)	0.21	0.35 (-4.07, 4.77)	0.88	-3.31 (-7.31, 0.69)	0.10
**Systolic blood pressure mmHg**	76	117.91 (11.81)	99	116.42 (12.50)	141	119.77 (72.55)	-1.39 (-4.83, 2.05)	0.43	2.38 (-0.78, 5.54)	0.14	-3.70 (-6.70, -0.70)	0.016
**Diastolic blood pressure mmHg**	76	72.91 (11.81)	99	70.18 (10.25)	141	72.55 (10.64)	-2.17 (- 4.93, 0.59)	0.12	0.28 (-2.28, 2.84)	0.83	-2.41 (-4.82, -0.01)	0.05
**Maternal Postnatal Outcomes at 3 months**												
**HbA1c mmol/mol**	59	36.76 (3.82)	79	36.23 (3.59)	98	37.20 (4.43)	-2.94 (-5.53, -0.35)	0.027	0.43 (-2.43, 3.29)	0.76	-3.37 (-6.03, -0.71)	0.014
**HbA1c %**	59	5.52 (0.35)	79	5.47 (0.33)	98	5.56 (0.41)	-0.27 (-0.51, -0.03)	0.027	0.04 (-0.22, 0.30)	0.76	-0.31 (-0.55, -0.07)	0.014
**TIR (3.9-10.0 mmol/l) %**	40	98.07 (3.16)	68	98.05 (3.08)	80	96.22 (6.61)	-0.15 (-2.30, 1.99)	0.89	-1.73 (-3.81, 0.36)	0.10	1.57 (-0.21, 3.35)	0.08
**CGM mean glucose mmol/l**	40	6.21 (0.58)	68	6.26 (0.67)	80	6.32 (0.87)	0.09 (-0.22, 0.39)	0.57	0.07 (-0.23, 0.36)	0.66	0.02 (-0.23, 0.27)	0.87
**CGM mean glucose mg/dL**	40	111.81 (10.48)	68	112.70 (12.10)	80	113.94 (15.69)	1.58 (-3.92, 7.07)	0.57	1.21 (-4.13, 6.55)	0.66	0.37 (-4.18, 4.91)	0.87
**Maternal weight kg**	63	84.54 (16.03)	82	91.79 (18.15)	104	86.90 (20.00)	-1.97 (-3.91, -0.03)	0.047	2.35 (0.54, 4.16)	0.011	-4.32 (-6.05, -2.60)	<0.001
**Maternal weight change from enrolment to 3 months**	63	-6.19 (5.34)	82	-8.54 (6.33)	104	-3.90 (5.41)	-2.20 (-4.10, -0.29)	0.024	2.33 (0.53, 4.15)	0.012	-4.53 (-6.23, -2.84)	<0.001
**Maternal BMI kg/m2**	63	31.60 (5.27)	82	33.71 (6.06)	104	32.71 (6.89)	-0.72 (-1.43, -0.02)	0.046	0.87 (0.21, 1.54)	0.010	-1.60 (-2.23, -0.97)	<0.001
**Maternal BMI change from enrolment to 3 months**	63	-2.32 (-10.44, 1.6)	82	-3.16 (-15.71, 1.83)	104	-1.48 (-9.38, 4.08)	-0.78 (-1.50, -0.07)	0.032	0.87 (0.20, 1.55)	0.012	-1.65 (-2.29, 1.02)	<0.001
**Systolic blood pressure mmHg**	60	116.45 (13.36)	78	119.91 (13.38)	97	119.24 (14.41)	5.16 (0.93, 9.40)	0.017	3.37 (-0.64, 7.38)	0.10	1.79 (-1.97, 5.56)	0.35
**Diastolic blood pressure mmHg**	60	80.57 (15.40)	78	80.00 (13.30)	97	79.30 (11.17)	0.80 (-3.44, 5.04)	0.71	-1.20 (-5.22, 2.81)	0.56	2.00 (-1.77, 5.78)	0.30

**SAFETY OUTCOMES**						
**Small for gestational age Intergrowth**	89	2(2.25)	114	3 (2.63)	157	10 (6.37)
**Stillbirth**	92	0 (0.00)	119	0 (0.00)	179	0 (0.00)
**Neonatal death**	92	0 (0.00)	119	0 (0.00)	179	0 (0.00)
**Maternal death**	92	0 (0.00)	119	0 (0.00)	179	0 (0.00)
**Congenital anomaly^**	88	0 (0.00)	114	1 (0.88)	158	1 (0.63)

^ In the weight gain group: bilateral blepharoptosis. In the weight loss group: congenital haemangioma.

Supplementary table 7: Food menu for participants. All individual meals and each complete snack pack contained 40% energy from carbohydrate, 35% from fat and 25% from protein. An additional vegetable/salad pack was provided if requested which added an additional 20-30 kcal per day and used seasonal ingredients.

**Breakfast (1 per day)**	**Main meals (2 per day)**	**Additional Items**
Cheese and Mushroom Omelette with rosti<br>Cheese and Ham Omelette with rosti<br>Breakfast bun (with egg bacon and mushroom filling)<br>Porridge with Jam and nuts<br>Granola<br>Cinnamon Porridge with Sunflower Seeds<br>Cherry and Almond Yoghurt<br>Blueberry Yoghurt<br>Welsh Rarebit<br>Seeded Bagel with Almond butter and Philadelphia<br>Scrambled Tofu with potato rosti<br>Scrambled Tofu and mushroom Breakfast bun	Fish Goujons<br>Salmon with Lemon Puy Lentils<br>Moroccan Spiced Chicken Wrap<br>Mushroom Stroganoff<br>Roasted Vegetable Lasagne<br>Vegetarian Chilli<br>Vegetarian Bean Stew<br>Vegetarian Spaghetti Bolognese<br>Macaroni Cheese with Kale<br>Aloo Mutter Paneer with Rice<br>Mexican Bean Enchilada<br>Chickpea and Spinach Curry with Spiced Paneer<br>Beef Lamb and Venison<br>Beef Madras<br>Chilli Con Carne<br>Beef in Black Bean sauce<br>Venison Sausages in Red Wine<br>Spaghetti Bolognese<br>Lamb Curry (medium)<br>Tandoori Chilli Chicken<br>Chicken Schnitzel<br>Thai Red Chicken Curry (hot)<br>Chicken Korma<br>Chicken Tikka Masala<br>Roast Turkey Dinner<br>Sausage and Pasta Bake<br>Pork Dijon	Snack Packs (1 per day)<br>Typical contents:<br>Several pieces of fruit<br>Savoury snacks -nuts, boiled eggs, crisps, veg sticks, hummus<br>Low sugar treat items – very dark chocolate, plain biscuit.<br><br>Vegetable/ Salad Pack (1 per week)<br>Typical contents:<br>Mushroom<br>Cauliflower<br>Courgette<br>Broccoli<br>Carrot<br>Little Gem<br>Cucumber<br>Carrot<br>Celery<br>Baby Plum tomatoes<br>Spring Onions<br>Radishes

List of Trial Steering Committee (TSC) Members

Prof Roy Taylor, Chair, consultant diabetologist, expertise in achieving type 2 diabetes remission in DiRECT study

Dr Adrian Park, consultant in obesity medicine

Prof Gordon Smith, consultant obstetrician, expertise in prediction of suboptimal birth outcomes.

Prof Helen Murphy, consultant diabetologist, expertise in interventional studies in pregnancy

Prof Vern Farewell, statistician.

Dr Angela Flynn, Senior nutritional scientist, expertise in diet in pregnancy

Dr Pam Dyson, diabetes dietician, expertise in nutritional management of diabetes

Ms Yvonne Nickerson, patient representative.

Ms Kamini Shah, Head of research funding, Diabetes UK.

Claire Meek, chief investigator

Other TSC attendees:

Deborah Hughes (DH)

Joanne Brown (JB)

Danielle Jones (DJ)

Laura Kusinski (LK)

List of Data Monitoring & Safety Board Members

Dr Rebecca Cannings-John, statistician with expertise in women and children's health.

Prof Rebecca Reynolds, consultant diabetologist, expertise in obesity in pregnancy

Prof Fionnuala MacAuliffe, consultant obstetrician, expertise in diabetes in pregnancy

List of Investigators (Listed in alphabetical order by institution.)

Dr Claire Meek; Cambridge University Hospitals NHS Foundation Trust

Rabia Zill-E-Huma; East and North Hertfordshire NHS Trust Lister

Lauren Smith; Kettering General Hospital NHS Foundation Trust

Dr Helen Murphy; Norfolk and Norwich University Hospitals NHS Foundation Trust

Dr Erick Leyva Caraballo; North West Anglia NHS foundation Trust Hinchingbrooke

Dr Erick Leyva Caraballo; North West Anglia NHS foundation Trust Peterborough

Salman Kidwal; Queen Elizabeth Hospital Kings Lynn NHS Foundation Trust

Mr Phillip Donkor; The Princess Alexandra Hospital Trust Harlow

List of Research Team Members

Coralie Glenn-Samsun

Lauren Andrews

Deborah Hughes

Jodie Carpenter

Kimberley Morris

Danielle Jones

Laura Kusinski

Suzanne Smith

Patrycja Tobolska

Joanne Brown

Elizabeth Turner

Tara Lee

Matilda Matthews

Laura Harris

Jo Brown

Edwina Lee

Joanne Finn

Hollie Curgenven

Sarah Johnson

Parizade Raymond

Anna William

Bincy Kariyadil

Inclusion criteria (from protocol version 11; 23/9/2020)

- **Women with gestational diabetes diagnosed before 30+6 weeks' gestation using a standard clinical 75g OGTT in accordance with the guidelines of the National Institute of Health and Care Excellence (NICE)(37).**
- The NICE criteria state that the diagnosis of gestational diabetes will be made with one or more glucose concentrations during the OGTT of:
  - ≥5.6 mmol/l in the fasting state
  - ≥7.8 mmol/l 2 hours after 75g glucose(38).

- During periods when antenatal OGTTs are suspended due to the restrictions caused by the Sars-CoV-2 virus, diagnosis of gestational diabetes will be made according to the criteria laid down at:

https://www.rcog.org.uk/globalassets/documents/guidelines/2020-07-10-guidance-for-maternal-medicine.pdf , namely HbA1c ≥ 39mmol/mol or Fasting Blood Glucose ≥ 5.6mmol/l or Random Blood Glucose ≥ 9mmol/l (para 3.2.2.1).

- **Overweight or obese (BMI ≥25 kg/m^2) at time of OGTT.**
- A ultrasound-confirmed viable singleton pregnancy
- Planned antenatal care at the same centre or a different study centre throughout their pregnancy (ie: not planning to move away from the region before delivery).

Exclusion criteria (from protocol version 11; 23/9/2020)

Women will be excluded if any of the following criteria apply:

- Evidence of multiple pregnancy on ultrasound
- Evidence of severe congenital anomaly on ultrasound
- Patient planning to terminate the pregnancy for any reason
- Significant pre-pregnancy diseases or comorbidities which increase risk in pregnancy, for example renal failure, severe liver disease, transplantation, cardiac failure, psychiatric conditions requiring in-patient admission (<1 year).
- Significant complications in the current pregnancy, such as threatened preterm labour, severe anaemia (Hb<8g/dl) or intra-uterine growth restriction (IUGR)
- Previous diagnosis of diabetes outside of pregnancy
- HbA1c at baseline of ≥48 mmol/mol.
- Medications at the time of the OGTT which may interfere with the results of the OGTT (for example, steroids, immunosuppressants, certain antipsychotics)
- Estimated fetal weight <10th percentile at diagnosis of gestational diabetes
- Maternal requirement for a highly specialised diet (e.g. vegan)
- Maternal severe food allergy, for example, a nut allergy causing anaphylaxis
- Weight loss of >5% pre-pregnancy weight during pregnancy, prior to 28 weeks.

Definitions of Trial Outcomes

Primary Outcomes:

Maternal weight change was defined as: weight at 36 weeks' gestation – weight at study enrolment (kg).

Neonatal standardised birthweight was calculated based on INTERGROWTH, but data are also presented as GROW customised centiles.

Selected Secondary Outcomes:

Large for gestational age was defined as birthweight $\geq$90th centile for gestational age.

Small for gestational age was defined as birthweight <10th centile for gestational age.

Neonatal hypoglycaemia was defined as a low blood glucose requiring intravenous dextrose

Estimated gestational age at birth was based on ultrasound assessment of gestational age.

Maternal glycaemia on continuous glucose monitoring time in range was defined as 3.5 – 6.7 mmol/l (63-120 mg/dl)
