# Supplementary material for: Reduced-energy diet in women with gestational diabetes: the dietary intervention in gestational diabetes DiGest randomized clinical trial
Source: Nat Med. 2025 Feb 19;31(2):514–23. doi: 10.1038/s41591-024-03356-1 (PMC11839452; doi:10.1038/s41591-024-03356-1)
Supplement: Supplementary file 2 — Reporting Summary [file 41591_2024_3356_MOESM2_ESM.pdf]

nature portfolio

Corresponding author(s): Prof Claire Meek

Last updated by author(s): Sep 25, 2024

Reporting Summary

Nature Portfolio wishes to improve the reproducibility of the work that we publish. This form provides structure for consistency and transparency in reporting. For further information on Nature Portfolio policies, see our Editorial Policies and the Editorial Policy Checklist.

Statistics

For all statistical analyses, confirm that the following items are present in the figure legend, table legend, main text, or Methods section.

n/a	Confirmed	
☐	☒	The exact sample size (*n*) for each experimental group/condition, given as a discrete number and unit of measurement
☐	☒	A statement on whether measurements were taken from distinct samples or whether the same sample was measured repeatedly
☐	☒	The statistical test(s) used AND whether they are one- or two-sided<br>*Only common tests should be described solely by name; describe more complex techniques in the Methods section.*
☐	☒	A description of all covariates tested
☐	☒	A description of any assumptions or corrections, such as tests of normality and adjustment for multiple comparisons
☐	☒	A full description of the statistical parameters including central tendency (e.g. means) or other basic estimates (e.g. regression coefficient) AND variation (e.g. standard deviation) or associated estimates of uncertainty (e.g. confidence intervals)
☒	☐	For null hypothesis testing, the test statistic (e.g. *F*, *t*, *r*) with confidence intervals, effect sizes, degrees of freedom and *P* value noted<br>*Give P values as exact values whenever suitable.*
☒	☐	For Bayesian analysis, information on the choice of priors and Markov chain Monte Carlo settings
☒	☐	For hierarchical and complex designs, identification of the appropriate level for tests and full reporting of outcomes
☒	☐	Estimates of effect sizes (e.g. Cohen's *d*, Pearson's *r*), indicating how they were calculated

Our web collection on statistics for biologists contains articles on many of the points above.

Software and code

Policy information about availability of computer code

Data collection	Database Castor (v2024.3.1.0). Randomisation was done using the library 'blockrand' in the statistical package R.
Data analysis	STATA (Version 17.0; StataCorp)

For manuscripts utilizing custom algorithms or software that are central to the research but not yet described in published literature, software must be made available to editors and reviewers. We strongly encourage code deposition in a community repository (e.g. GitHub). See the Nature Portfolio guidelines for submitting code & software for further information.

Data

Policy information about availability of data

All manuscripts must include a data availability statement. This statement should provide the following information, where applicable:

- Accession codes, unique identifiers, or web links for publicly available datasets
- A description of any restrictions on data availability
- For clinical datasets or third party data, please ensure that the statement adheres to our policy

Anonymised individual participant data is available upon request from the corresponding author, subject to approval from trial steering groups. Data will not be uploaded to a repository in advance of publication due to the potential for subject identification.

Research involving human participants, their data, or biological material

Policy information about studies with human participants or human data. See also policy information about sex, gender (identity/presentation), and sexual orientation and race, ethnicity and racism.

Reporting on sex and gender	This study included pregnant individuals who were assigned female sex at birth. We did not exclude pregnant people based upon gender at the time of recruitment. For infants, we collected information on neonatal sex from medical records.
Reporting on race, ethnicity, or other socially relevant groupings	We recruited participants to this study regardless of age, gender, religion, ethnicity or political views. Ethnicity is detailed in table 1 of the manuscript.
Population characteristics	Maternal age, BMI, ethnicity, primiparous, maternal education, index of multiple deprivation decile, previous diabetes in pregnancy, smoking, physical activity, habitual energy intake, basal metabolic rate, systolic blood pressure, diastolic blood pressure, gestational age at diagnosis, medication use, OGTT results and HbA1c results.
Recruitment	Women aged >18 years old with an ultrasound-confirmed singleton pregnancy, gestational diabetes diagnosed before 30+6 weeks' gestation and a BMI >25 kg/m2 were recruited to the trial. The diagnosis of gestational diabetes was based on the criteria of the National Institute for Health and Care Excellence (NICE; 75g OGTT >5.6 mmol/L (>100mg/dL) fasting and >7.8 mmol/L (>140mg/dL) at 2 hours; previous gestational diabetes: glucometer testing recurrently above targets fasting >5.3 mmol/L fasting and >7.8 mmol/L 1 hour post meal). Women were assessed for eligibility by the research team and approached with the patient information sheet.
Ethics oversight	The protocol was approved by the National Research Ethics Committee, UK (reference 18/WM/0191) and the NHS Health Research Authority (IRAS 242924; ISRCTN 65152174)

Note that full information on the approval of the study protocol must also be provided in the manuscript.

Field-specific reporting

Please select the one below that is the best fit for your research. If you are not sure, read the appropriate sections before making your selection.

☒ Life sciences ☐ Behavioural & social sciences ☐ Ecological, evolutionary & environmental sciences

For a reference copy of the document with all sections, see nature.com/documents/nr-reporting-summary-flat.pdf

Life sciences study design

All studies must disclose on these points even when the disclosure is negative.

Sample size	The original sample size was n=500 which provided >90% power to identify a 0.33 SD (1kg) difference in maternal weight change between groups (maternal primary outcome) and >90% power for identification of a 0.3 SD (150g) difference in standardised birthweight (neonatal primary outcome), allowing for 20% withdrawals, with a significance level of 5% for each of the two primary outcomes (two-sided). However, in May 2022, the data safety monitoring board (DMSB) recommended reducing the sample size to 380 following an interim analysis after 250 participants were recruited. Using the data collected to that stage, the probability of finding the original effect size was calculated to be 0.72 if 380 women were recruited and 0.85 for both outcomes if 500 women were recruited. The DMSB therefore considered that the trial should not be stopped for futility after n=250, but that 380 participants was sufficient to identify if significant differences were present. These recommendations were peer reviewed prior to implementation. We monitored withdrawal rates during the trial prior to the collection of primary endpoint data (11%) and therefore recruited 428 women to ensure there was primary outcome information available for 380 pregnancies.
Data exclusions	The Missing Indicator Method (MIM) was used to assess the potential impact of missing data on effect estimation. Multiple imputation was used to investigate the impact of missing data on the intervention effect for the primary outcomes, assuming data were missing at random. Three participants withdrew after visit 1 but before randomisation, their data was excluded from the baseline data shown in Table 1 and subsequent analysis.
Replication	N/A
Randomization	The randomisation protocol was designed in advance by one of the study statisticians (VF). The allocations were programmed into the food ordering website, to ensure participants were automatically randomised while maintaining blinding. Randomisation was implemented using the library 'blockrand' in the statistical package R. The randomisation was done in permuted blocks of size 6, in a 1:1 ratio and stratified by centre.
Blinding	We chose to use a whole-diet intervention to reduce bias, facilitate blinding and reduce socioeconomic, educational and cultural barriers to dietary adherence. The allocations were programmed into the food ordering website, to ensure participants were automatically randomised while maintaining blinding. Both participants and study team were blinded to the data until permission from the Trial Steering Committee was given after all participants had given birth to their babies.

Reporting for specific materials, systems and methods

We require information from authors about some types of materials, experimental systems and methods used in many studies. Here, indicate whether each material, system or method listed is relevant to your study. If you are not sure if a list item applies to your research, read the appropriate section before selecting a response.

Materials & experimental systems

n/a	Involved in the study
☒	☐ Antibodies
☒	☐ Eukaryotic cell lines
☒	☐ Palaeontology and archaeology
☒	☐ Animals and other organisms
☐	☒ Clinical data
☒	☐ Dual use research of concern
☒	☐ Plants

Methods

n/a	Involved in the study
☒	☐ ChIP-seq
☒	☐ Flow cytometry
☒	☐ MRI-based neuroimaging

Clinical data

Policy information about clinical studies

All manuscripts should comply with the ICMJE guidelines for publication of clinical research and a completed CONSORT checklist must be included with all submissions.

Clinical trial registration: ISRCTN 65152174

Study protocol: Kusinski LC, Murphy HR, De Lucia Rolfe E, Rennie KL, Oude Griep LM, Hughes D, et al. Dietary Intervention in Pregnant Women with Gestational Diabetes; Protocol for the DiGest Randomised Controlled Trial. Nutrients. 2020;12(4).

Data collection: The DiGest trial was a randomised, controlled, double-blind, whole-diet intervention study with a parallel design conducted in eight hospital centres in England. These include; Cambridge University Hospitals NHS Foundation Trust, East and North Hertfordshire NHS Trust Lister, Kettering General Hospital NHS Foundation Trust, Norfolk and Norwich University Hospitals NHS Foundation Trust, North West Anglia NHS foundation Trust Hinchingbrooke, North West Anglia NHS foundation Trust Peterborough, Queen Elizabeth Hospital Kings Lynn NHS Foundation Trust, The Princess Alexandra Hospital Trust Harlow. Recruitment of the study ran from November 2019 to July 2023. The participants were recruited and assessed in the hospital setting but also given the option to have some of their visits at home due to the Covid-19 pandemic.

Outcomes: The DiGest trial has two co-primary endpoints; maternal weight change between enrolment and 36 weeks gestation and neonatal birthweight, assessed using neonatal sex-appropriate SD scores (SDS), calculated for weight and length measurements (with adjustment for gestational age at birth) using customised centiles. Maternal weight was measured a member of the research team using body weight scales. Neonates were measured at birth by the clinical team using local hospital procedures.
Secondary maternal outcomes include maternal weight, BMI, glycaemia (using CGM metrics as per the international time-in-range consensus recommendations, HbA1c, cardiometabolic health (blood pressure, lipids, fasting insulin, fasting glucose), maternal food choice and eating behaviour, quality of life, treatments administered for gestational diabetes, birth modality / complications.
Secondary neonatal outcomes include gestational age at delivery, preterm delivery (<37 weeks), large/small- for-gestational age, cord blood C-peptide, admission to the neonatal intensive care unit (NICU), neonatal jaundice requiring phototherapy, Apgar scores, anthropometry, neonatal hypoglycaemia (defined as a capillary glucose <2.6 mmol/L on one or more occasions, within the first 48 hours of life starting at least 30 minutes after birth, and necessitating treatment either with 40% glucose gel administered to the buccal mucosa and/or with intravenous dextrose), neonatal nasogastric feeding and feeding type on discharge from hospital. Infant feeding choices and feeding history will also be examined at 3 months postpartum.

Plants

Seed stocks: N/A

Novel plant genotypes: N/A

Authentication: N/A
